# Repression of Transcription at DNA Breaks Requires Cohesin throughout Interphase and Prevents Genome Instability

**DOI:** 10.1016/j.molcel.2018.11.001

**Published:** 2019-01-17

**Authors:** Cornelia Meisenberg, Sarah I. Pinder, Suzanna R. Hopkins, Sarah K. Wooller, Graeme Benstead-Hume, Frances M.G. Pearl, Penny A. Jeggo, Jessica A. Downs

**Affiliations:** 1 Epigenetics and Genome Stability Team, The Institute of Cancer Research, 237 Fulham Road, London SW3 6JB, UK; 2 Bioinformatics Group, School of Life Sciences, University of Sussex, Falmer, Brighton BN1 9QJ, UK; 3 Genome Damage and Stability Centre, University of Sussex, Falmer, Brighton BN1 9RQ, UK

**Keywords:** SA2, STAG2, PBAF, PBRM1, SMARCA4, DNA repair, transcriptional silencing, SWI/SNF, cancer

## Abstract

Cohesin subunits are frequently mutated in cancer, but how they function as tumor suppressors is unknown. Cohesin mediates sister chromatid cohesion, but this is not always perturbed in cancer cells. Here, we identify a previously unknown role for cohesin. We find that cohesin is required to repress transcription at DNA double-strand breaks (DSBs). Notably, cohesin represses transcription at DSBs throughout interphase, indicating that this is distinct from its known role in mediating DNA repair through sister chromatid cohesion. We identified a cancer-associated SA2 mutation that supports sister chromatid cohesion but is unable to repress transcription at DSBs. We further show that failure to repress transcription at DSBs leads to large-scale genome rearrangements. Cancer samples lacking SA2 display mutational patterns consistent with loss of this pathway. These findings uncover a new function for cohesin that provides insights into its frequent loss in cancer.

## Highlights

• Cohesin and PBAF repress gene transcription near DNA double-strand breaks (DSBs)
• Transcriptional repression at DSBs requires cohesin and PBAF throughout interphase
• Loss of DSB-induced gene repression leads to more large-scale genome alterations

## Graphical Abstract

**Figure:**
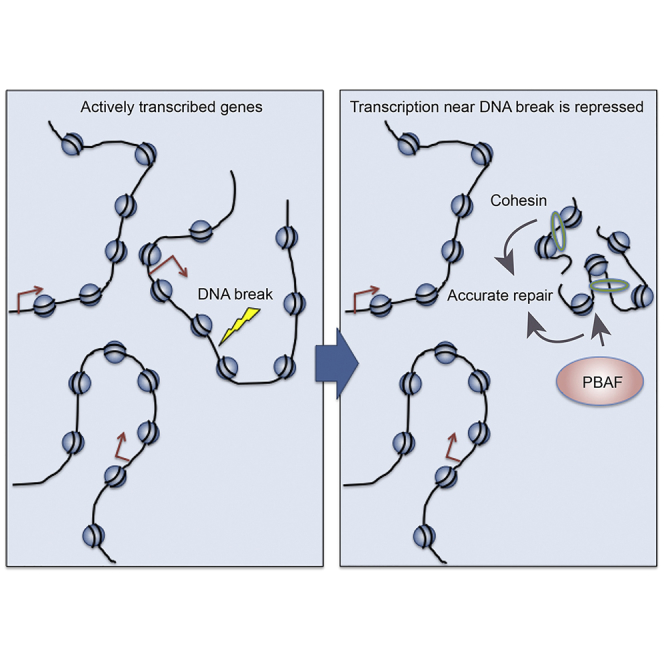

## Teaser

Meisenberg et al. show that, in response to DNA double strand breaks (DSBs), cohesin is required to repress nearby transcription. This happens throughout interphase, regardless of whether a sister chromatid is present. Failure to repress transcription near a DNA DSB results in an increase in large-scale chromosome rearrangements.

## Introduction

The cohesin complex is comprised of the core subunits SMC1A, SMC3, RAD21, and either SA1 (STAG1) or SA2 (STAG2). When bound to chromatin, cohesin associates with either the PDS5A or PDS5B regulatory subunits. Following DNA replication, the complex undergoes a transition along chromosome arms to establish sister chromatid cohesion, which is dependent on the acetyltransferases ESCO1 or ESCO2 (Dorsett and Ström, 2012). Cohesion is promoted by the association of sororin with acetylated SMC3, which prevents WAPL-mediated cohesin removal (Ladurner et al., 2016). In mammalian cells, it has been established that there is a division of labor between the cohesin complexes working at different chromosomal locations. At centromeres, cohesin complexes specifically contain SA2 and PDS5B, and depend on ESCO2 for establishment of cohesion (Canudas and Smith, 2009, Carretero et al., 2013, Remeseiro et al., 2012, Whelan et al., 2012).

Mutation of genes encoding cohesin subunits, such as SA2, is frequently observed in cancer. One obvious mechanism by which cohesin could function as a tumor suppressor is through preventing defects in chromosome segregation, which lead to aneuploidy, by maintaining normal sister chromatid cohesion. However, many cancers with mutations in SA2 do not display obvious aneuploidy (Hill et al., 2016). In addition to its role mediating sister chromatid cohesion, the cohesin complex is also able to regulate transcriptional activity (Losada, 2014) and is recruited to double-strand breaks (DSBs) to promote repair in S and G2 phases of the cell cycle (Dorsett and Ström, 2012). Either or both of these activities could contribute to the tumor suppressor activity of cohesin, but it is not yet clear whether or to what degree they do.

In response to a DNA DSB, cells respond by repressing transcription in the flanking chromatin (Shanbhag et al., 2010). This pathway is dependent on ATM (Iannelli et al., 2017, Shanbhag et al., 2010, Ui et al., 2015), and we found that the PBAF chromatin remodeling complex, one of two mammalian SWI/SNF complexes, is also important for this activity and functions downstream of ataxia telangiectasia mutated (ATM) (Kakarougkas et al., 2014).

Previously, we found that PBAF promotes sister chromatid cohesion at centromeres (Brownlee et al., 2014). Because both PBAF and cohesin are known to be recruited to DNA DSBs, we hypothesized that cohesin may play a role together with PBAF in repressing nearby transcription. Here, we show that it does. Notably, we find that cohesin is required for this pathway both in G1 and G2, demonstrating that this is a distinct function from its known role in promoting DNA repair through sister chromatid cohesion (Dorsett and Ström, 2012, Gelot et al., 2016). In addition, we provide evidence that the role of cohesin in repressing transcription contributes to the maintenance of genome stability through preventing large-scale genome rearrangements. Together, these findings reveal a new function for the cohesin complex in the cellular response to DNA DSBs that sheds light on its role in tumorigenesis.

## Results

### Cohesin Contributes to Transcriptional Repression at DNA DSBs

**Figure 1:**
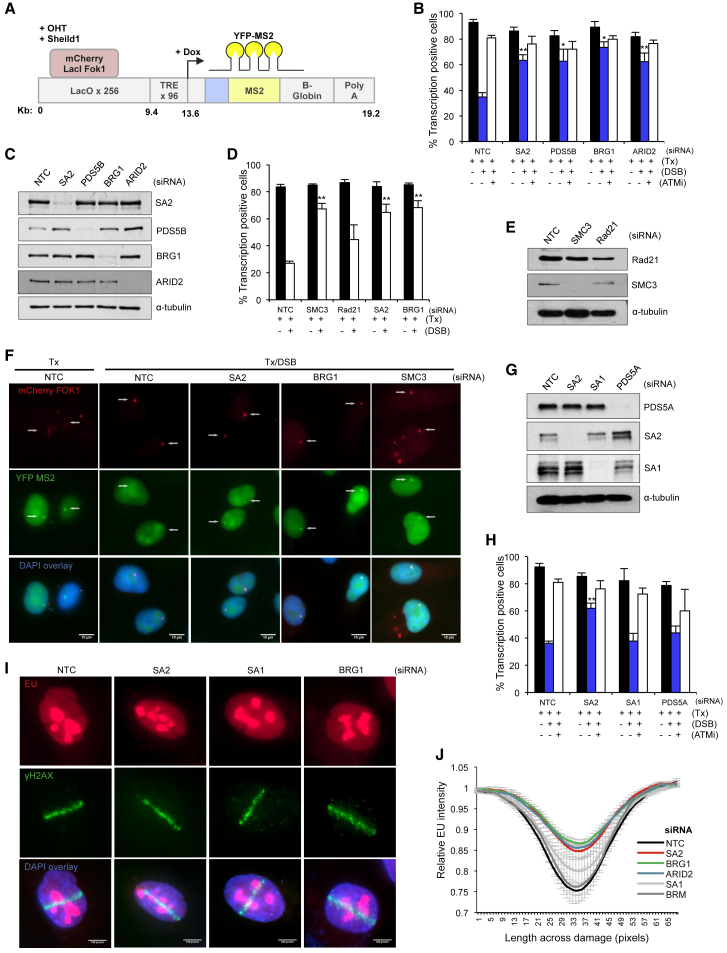
Cohesin Contributes to Transcriptional Repression at DNA Double-Strand Breaks (A) Cartoon of reporter construct (Tang et al., 2013) in which induction of the mCherry-tagged *Fok*I endonuclease results in double-strand break (DSB) induction in a region upstream of a doxycycline-inducible reporter gene. Ongoing transcription of the reporter gene can be visualized by the presence of a YFP-MS2 fusion protein that binds stem-loop structures in the nascent transcript. (B, D, and H) Quantification of ongoing transcription in U2OS reporter cells (263 IFII) treated with small interfering RNA (siRNA) targeting SA2, PDS5B, BRG1, or ARID2 (B), SMC3, Rad21, SA2, or BRG1 (D), or SA2, SA1, or PDS5A (H). NTC, non-targeting control. After addition of doxycycline to induce transcription (Tx), transcriptional repression was monitored in cells with or without induction of the *Fok*I endonuclease (DSB) by quantification of YFP-positive cells. Cells treated with 10 μM ATM inhibitor are indicated (ATMi). 150 cells were analyzed per condition per repeat. Data are presented as the mean ± SD; n = 4 (B), n = 3 (D and H). ^∗^p < 0.05, ^∗∗^p < 0.01 using Student’s t test. (C, E, and G) Western blot analysis of whole-cell extracts prepared from cells treated with siRNA targeting SA2, PDS5B, BRG1, or ARID2 (C), SMC3 or Rad21 (E), or SA2, SA1, or PDS5A (G). NTC, non-targeting control. α-Tubulin was used as a loading control. (F) Representative images of U2OS reporter cells analyzed in (B). Arrow indicates location of *Fok*I-induced DSB (mCherry) and/or YFP-MS2 transcript. (I) Representative images of cells assayed for transcriptional activity by monitoring EU incorporation after DNA damage induced by laser microirradiation and treatment with the indicated siRNA. (J) Quantification of EU signal (new mRNA synthesis) across the path of laser micro-irradiation in cells treated as in (I). Data are presented as mean ± SEM. A minimum of 30 cells were analyzed per repeat (n = 3–5 biological repeats). See also Figure S1.

To test whether cohesin is important for transcriptional repression in response to DNA breaks, we used an elegant reporter cell line developed by Greenberg and colleagues in which DSBs can be induced at a defined chromosomal location upstream of an inducible reporter gene (Tang et al., 2013; Figure 1A). Ongoing transcription can be visualized by the presence of a YFP-MS2 fusion protein that binds stem-loop structures in the nascent transcript, and DNA DSBs are introduced by induction of an mCherry-tagged *Fok*I endonuclease construct that is targeted to a region upstream of the promoter (Shanbhag et al., 2010, Tang et al., 2013).

Consistent with previous results, we found that transcriptional repression in response to DSBs is dependent on ATM and BRG1, which is the catalytic subunit of the PBAF chromatin remodeling complex (Figures 1B, 1C, and S1; Kakarougkas et al., 2014, Shanbhag et al., 2010). We also found that depletion of the PBAF subunit BAF200 (ARID2) leads to a similar defect (Figures 1B and 1C).

We tested the role of cohesin in this pathway and find that depletion of the core cohesin subunits SMC3 or RAD21 leads to defects in the ability of cells to repress transcription following DNA damage (Figures 1D and 1E). In addition, we find that depletion of SA2 and PDS5B, but not SA1 or PDS5A, leads to similar defects (Figures 1B–1H).

To analyze this response using a different approach, we monitored the incorporation of 5-ethynyl uridine (EU) following laser microirradiation induced damage as a measure of ongoing transcription (Figures 1I and S1). In control cells, quantification shows a reduction in EU signal in damaged chromatin (Figure 1J). Consistent with the known role in this pathway, loss of either the BRG1 or ARID2 subunits of PBAF leads to a change in this pattern that reflects more residual ongoing transcription (Figures 1I, 1J, and S1). In contrast, depletion of BRM, which is a subunit of the BAF remodeling complex, has no effect on this pathway (Figure 1J), suggesting that repression depends specifically on PBAF. Similarly, depletion of SA2, but not SA1, results in more EU incorporation in damaged chromatin than the control cells (Figures 1I and 1J).

The ATM-dependent pathway leading to transcriptional repression results in the accumulation of H2A K119 ubiquitination at sites of DNA damage (Shanbhag et al., 2010). Consistently, we found PBAF is required for H2A K119ub after irradiation (Kakarougkas et al., 2014; Figure S1E). We found that irradiation-induced H2A K119ub is impaired following depletion of SA2, but not SA1 (Figure S1E). We previously found that there is a small but reproducible increase in γH2AX foci at early time points following irradiation in the absence of PBAF subunits (Kakarougkas et al., 2014), and here, we found a similar increase in the absence of SA2, whereas SA1 depletion had no effect (Figure S1F). Together, these data demonstrate that the centromere-specific cohesin complex subunits are important for mediating transcriptional repression in response to DNA DSBs, and they suggest that it is functioning in the same pathway as PBAF.

### Transcriptional Repression near DNA Breaks Is Dependent on PBAF and Cohesin in Both the G1 and G2 Phases of the Cell Cycle

Cohesin is known to facilitate repair by homologous recombination (Dorsett and Ström, 2012, Losada, 2014). In addition, there is evidence that it promotes accurate non-homologous end joining (NHEJ) through use of the sister chromatid (Gelot et al., 2016). Because these repair activities involve the sister chromatid, we wondered whether the requirement for cohesin in mediating transcriptional repression in response to a DNA DSB was also restricted to the late S and G2 phases of the cell cycle when a sister chromatid is present.

**Figure 2:**
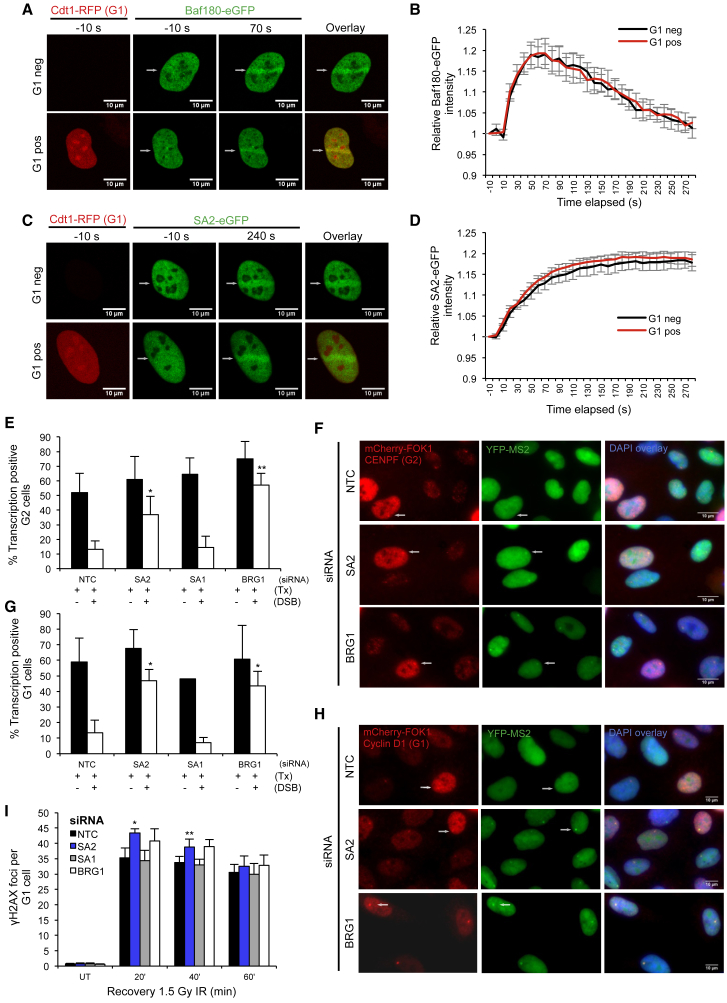
Cohesin- and PBAF-Dependent Transcriptional Repression at DNA Double-Strand Breaks Occurs in Both G1 and G2 Phases (A and C) Representative images of cells expressing GFP-BAF180 (A), GFP-SA2 (C), or Cdt1-RFP (to identify G1 phase cells) as indicated following laser microirradiation. (B and D) Quantification of GFP-BAF180 (B) or GFP-SA2 (D) recruitment to laser-microirradiation-induced damage in G1 cells (Cdt1-RFP positive) or cells outside of G1 (Cdt1-RFP negative). Data represent the relative mean signal intensity ± SEM for n = 6 (B) or n = 7 (D) biological repeats. At least 42 cells were analyzed in total for each construct per cell-cycle phase. (E) Quantification of transcription in CENPF-positive (G2 phase) U2OS reporter cells treated with the indicated siRNA (NTC, non-targeting control) with or without induction of the *Fok*I endonuclease. Data are presented as the mean ± SD. More than 40 cells were analyzed per condition per repeat (n = 3 biological repeats). (F) Representative images of U2OS reporter cells analyzed in (E). (G) Quantification of transcription in cyclin-D1-positive (G1 phase) U2OS reporter cells treated with the indicated siRNAs. Data are presented as the mean ± SD. More than 40 cells were analyzed per condition per repeat (n = 3 biological repeats). (H) Representative images of U2OS reporter cells analyzed in (G). (I) Quantification of γH2AX foci clearance following exposure to 1.5 Gy IR in G1 phase (cyclin D1 positive) U2OS cells treated with the indicated siRNA. Data are presented as mean ± SD; n = 3 biological repeats. ^∗^p < 0.05, ^∗∗^p < 0.01 using paired Student’s t test. See also Figure S2.

To investigate this, we first established that both SA2 and BAF180 are recruited to laser-induced microirradiation (Figures S2B–S2E). They appear to be independently recruited, as depletion of BAF180 does not impact on SA2 accumulation and vice versa (Figures S2J and S2K). Next, we examined whether these proteins are recruited to damaged chromatin in different cell-cycle stages by monitoring expression of an RFP-tagged Cdt1 construct (Figure S2A), which is expressed specifically in G1 phase (Sakaue-Sawano et al., 2008). Recruitment of BAF180 to laser induced microirradiation was the same in both G1-positive cells and cells outside of G1 (Figures 2A and 2B). Consistent with a previous report (Caron et al., 2012), we also found that the pattern of SA2 recruitment to damaged DNA was similar in cells both in and outside of G1 (Figures 2C and 2D), supporting the notion that cohesin is recruited to DSBs throughout interphase.

We then depleted PBAF and cohesin subunits and tested their ability to repress transcription in response to a DSB in either G1 or G2 phase cells. Cell-cycle stage was monitored using CENPF expression to identify cells in G2 and Cyclin D1 expression to identify G1 positive cells. As expected in G2 cells, we found that depletion of SA2 and BRG1 (but not depletion of SA1) led to a loss of DSB-induced repression (Figures 2E and 2F). Importantly, we found that both SA2 and BRG1 are also required for this activity in G1 cells (Figures 2G and 2H). In support of this, we also find that there is a reproducible increase in the number of γH2AX foci in G1 cells at early time points following irradiation when SA2 or BRG1 are depleted (Figure 2I).

These data demonstrate that the function of cohesin in the transcriptional response to DNA DSBs is distinct from its known role in promoting DNA repair through sister chromatid cohesion.

### Cohesion Establishment and Cohesin Loading Factors Are Important for Transcriptional Repression at DNA DSBs in Both G1 and G2 Phases

**Figure 3:**
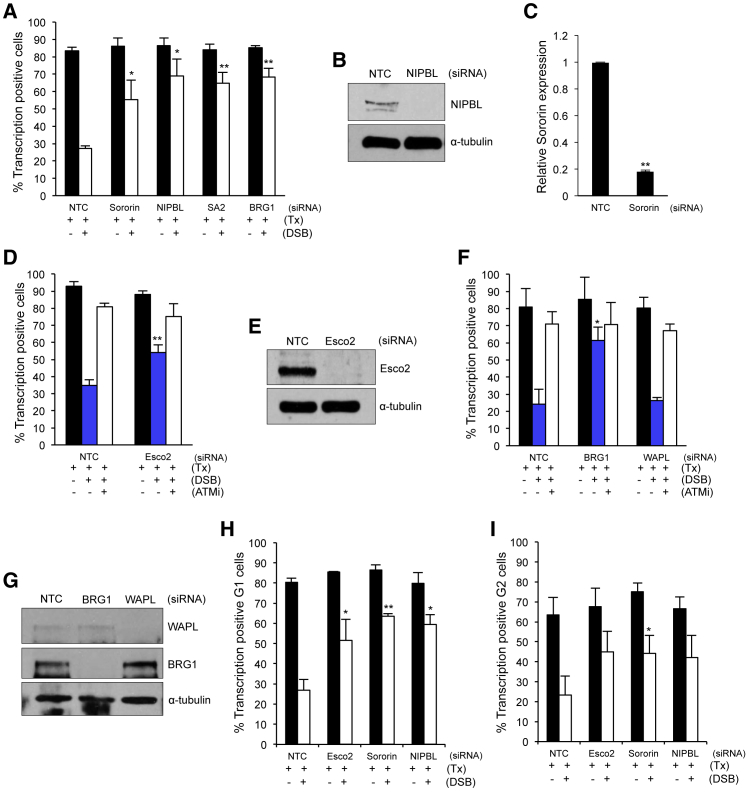
Cohesin Establishment and Loading Factors Are Important for Transcriptional Repression at DNA DSBs in Both G1 and G2 Phase Cells (A, D, and F) Quantification of transcription in asynchronous U2OS reporter cells with or without induction of the *Fok*I endonuclease (DSB) treated with siRNA targeting Sororin, NIPBL, SA2, or BRG1 (A), Esco2 (D), BRG1 or WAPL (F), and/or with 10 μM ATM inhibitor (NTC, non-targeting control). 150 cells were analyzed per condition, per repeat. Data are presented as mean ± SD; n = 3 (A), n = 4 (D), n = 4 (F) biological repeats. (B, E, and G) Western blot analysis of whole-cell extracts prepared from cells treated with siRNA targeting NIPBL (B), Esco2 (E), or BRG1 or WAPL (G). NTC, non-targeting control. α-Tubulin was used as a loading control. (C) qRT-PCR analysis of Sororin mRNA levels following siNTC or siSororin treatment to provide an indication of depletion efficiency. (H) Quantification of transcription in cyclin-D1-positive (G1 phase) U2OS reporter cells treated with the indicated siRNAs. Data are presented as the mean ± SD; n = 3 biological repeats. (I) Quantification of transcription in CENPF-positive (G2 phase) U2OS reporter cells treated with the indicated siRNA with or without induction of the *Fok*I endonuclease (DSB). More than 110 cells were analyzed per condition, per repeat. Data are presented as mean ± SD, n = 3 biological repeats. ^∗^p < 0.05, ^∗∗^p < 0.01 using paired Student’s t test. See also Figure S3.

We further investigated the genetic requirements related to cohesin function for this pathway. The recruitment of cohesin to sites of DNA damage (Figure 2) suggests that the cohesin loader might be involved, and it has recently been shown that the NIPBL cohesin loader is recruited to DNA damage throughout the cell cycle (Bot et al., 2017). We find that depletion of the cohesin loader NIPBL leads to a defect in the ability of cells to repress transcription at damaged DNA (Figures 3A, 3B, and S3C).

Notably, we also find that Sororin and ESCO2 are required for transcriptional repression in response to a DNA break and display a defect similar to that observed when either SA2 or PBAF subunits are depleted (Figures 3A–3E). In contrast, we find that depletion of the negative regulator of cohesion, WAPL, has no detectable impact on this pathway (Figures 3F and 3G). We also found no effect on this pathway when CTCF was depleted (Figures S3A and S3B).

We next investigated whether these cohesin loading and establishment factors are also required outside of S and G2 phases when no sister chromatid is present, and we found that similar to the requirement for cohesin subunits, they are required for the ability to repress transcription in response to DNA damage in both G1 and G2 phase cells (Figures 3H and 3I).

### A Cancer-Associated Mutant of SA2 that Is Proficient for Sister Chromatid Cohesion Is Not Able to Repress Transcription at DNA DSBs

It has been shown that mutation of the SA2 encoding gene (STAG2) in cancer is not always associated with aneuploidy (Balbás-Martínez et al., 2013), suggesting that in at least some cases, there is no defect in chromosome segregation as a result of mutations in cohesin. In support of this, a recent mechanistic study showed that a subset of cancer-associated SA2 mutations are proficient in mediating sister chromatid cohesion and chromosome segregation (Kim et al., 2016). We tested the possibility that these mutants might not be able to repress transcription following DSBs.

**Figure 4:**
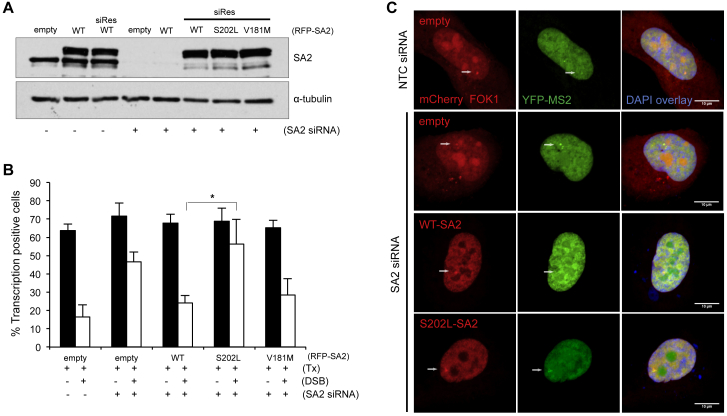
A Sister Chromatid Cohesion-Proficient Cancer-Associated SA2 Mutant Is Not Able to Support Transcriptional Repression at DNA DSBs (A) Western blot analysis of SA2 in whole-cell extracts prepared from cells following siRNA depletion (where indicated) and transfection with siRNA-sensitive or siRNA-resistant (siRes) SA2 constructs. (B) Quantification of transcription in U2OS reporter cells treated with siSA2 and transfected with the indicated siRNA-resistant SA2 construct with or without induction of the *Fok*I endonuclease (DSB). 100 cells were analyzed per condition per repeat. Data represent the mean ± SD; n = 3 biological repeats. ^∗^p < 0.05, ^∗∗^p< 0.01 using paired Student’s t test. (C) Representative images of U2OS reporter cells analyzed in (B). See also Figure S4.

To do this, we chose to study two point mutations that had no obvious impact on protein stability, interaction with other cohesin subunits, or sister chromatid cohesion (Kim et al., 2016). These were V181M, identified in a myeloid leukemia, and S202L from a bladder cancer. We introduced the mutations into a GFP-tagged siRNA-resistant SA2 expression construct (Figure S4). When we transfected the mutant or wild-type expression constructs into cells that were depleted of endogenous SA2 (Figure 4A), we found that the wild-type and V181M constructs were able to rescue transcriptional repression after DSB induction (Figures 4B and 4C). In contrast, the S202L mutant construct was unable to rescue DNA DSB-induced transcriptional repression (Figures 4B and 4C). These data identify SA2-S202L as a separation of function mutation and raise the possibility that this pathway might be important for the ability of cohesin, and particularly SA2, to act as a tumor suppressor.

### PBAF and Cohesin Repress Large-Scale Chromosome Rearrangements between Actively Transcribed Genes

If transcriptional repression at DSBs is important for preventing tumorigenesis, it suggests that this pathway in some way prevents genome instability. As a consequence of their three-dimensional genome organization as well as their topological and chromatin environment, actively transcribed genes are vulnerable to translocations and large-scale genome rearrangements (Osborne, 2014). Consequently, one possible function of transcriptional repression following DSB induction is to prevent mis-rejoining of DSBs within active genes leading to large-scale genome rearrangements.

**Figure 5:**
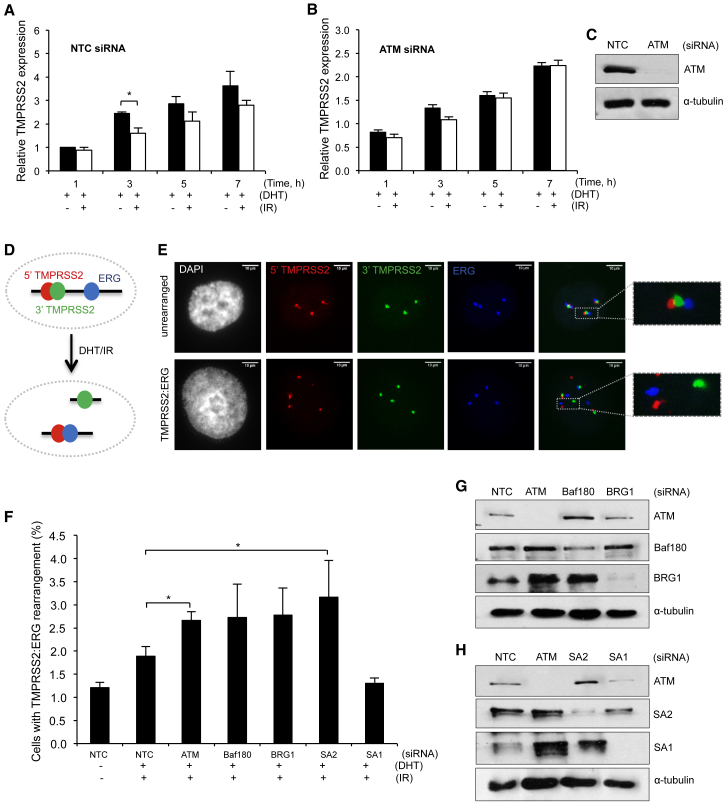
Depletion of Cohesin or PBAF Leads to Increased Chromosome Rearrangements in the TMPRSS2 Gene Following Transcriptional Induction and DNA Damage (A and B) qRT-PCR analysis of relative TMPRSS2 transcript levels in LNCaP cells following transcriptional induction with 300 nM DHT (+DHT) and with or without 10 Gy irradiation after siRNA depletion with non-targeting control (NTC; A) or ATM (B). Data are presented as the mean ± SEM; n = 3 biological repeats. (C) Western blot analysis of whole-cell extracts prepared from cells treated with siNTC or siATM. α-Tubulin was used as a loading control. (D) Cartoon of gene organization and location of probes used in FISH assays to monitor TMPRSS2:ERG translocations. Following treatment with DHT and IR, LNCaP cells underwent frequent rearrangements, as illustrated. (E) Representative FISH images showing cells with (bottom) and without (top) TMPRSS2:ERG translocations (see D). (F) Analysis of translocations between TMPRSS2 and ERG in LNCaP cells by FISH following transcription activation and DNA DSB induction in cells treated with the indicated siRNA. 50 cells were analyzed per condition per repeat. Data are presented as the mean ± SEM, and a minimum of 3 (up to 12) biological repeats were performed for each condition. (G and H) Western blot analysis of whole-cell extracts prepared from LNCaP cells treated with siRNA targeting ATM, BAF180, or BRG1 (G) or ATM, SA2, or SA1 (H). NTC, non-targeting control. α-Tubulin was used as a loading control. ^∗^p < 0.05, ^∗∗^p < 0.01 using unpaired Student’s t test. See also Figure S5.

To test the hypothesis that repression of transcription at DNA breaks is important for the fidelity of repair, we set out to monitor translocations between actively transcribed genes in a physiologically relevant system. For these studies, we chose the prostate cancer cell line LNCaP, in which the androgen-responsive gene TMPRSS2 and the ERG gene have been shown to undergo translocations in an androgen- and DNA-damage-responsive manner (Lin et al., 2009; Figures 5D and 5E), and this translocation event is frequently present in prostate cancers (Tomlins et al., 2005). We find that androgen-induced TMPRSS2 transcription is not substantially impaired in the absence of either BAF180 or SA2 (Figures S5A and S5B), making this a good system to investigate the DSB-induced transcriptional repression pathway.

We began by investigating whether androgen-induced expression of the TMPRSS2 gene is repressed after DNA damage. First, we compared the expression levels in cells treated with 5α-dihydrotestosterone (DHT) to activate transcription of TMPRSS2 and compared this to DHT-treated cells that were also irradiated. Treatment with ionizing radiation (IR) has previously been shown to result in the accumulation of DSBs at hotspots within the TMPRSS2 gene (Lin et al., 2009). These DSBs are not directly produced by IR treatment but arise indirectly in a damage- and androgen-dependent manner (Lin et al., 2009). We found that transcription levels were lower in irradiated cells than in unirradiated controls following induction with DHT (Figure 5A), consistent with transcriptional repression following DSB induction. We also monitored transcription in cells treated with DHT for 16 hr to reach steady-state transcription levels and then irradiated the cells. Again, we found that transcription levels decreased in response to IR, consistent with DSB-induced transcriptional silencing (Figure S5C). Additionally, we monitored transcriptional activity in cells after depletion of ATM and found that expression levels after damage remained the same as in undamaged cells, consistent with a role for ATM in DSB-induced transcriptional silencing (Figures 5B and 5C).

Next, we investigated the impact of loss of this pathway on the formation of translocations between the TMPRSS2 and ERG genes. We monitored translocation formation using a fluorescence *in situ* hybridization (FISH)-based assay (Fernández-Serra et al., 2013; Figures 5D and 5E) or using qRT-PCR (Figures S5D–S5F), and found, consistent with previous reports, an increase after treating cells with DHT and IR (Figures 5F and S5D). Notably, we found the number of translocations is further increased when we depleted cells of ATM, the PBAF subunits BAF180 or BRG1, or SA2 (Figures 5F–5H and S5D–S5F). In contrast, depletion of SA1 did not lead to an increase in translocation frequency (Figures 5F and 5H). These data support the idea that the transcriptional repression of genes in the vicinity of DNA breaks functions to prevent mis-rejoining of the broken DNA ends and thus prevent genome rearrangements.

### PBAF and Cohesin Are Important for Preventing Chromosome Rearrangements in G1 Phase Cells, Specifically When DSBs Are near Strong Transcriptional Activity

**Figure 6:**
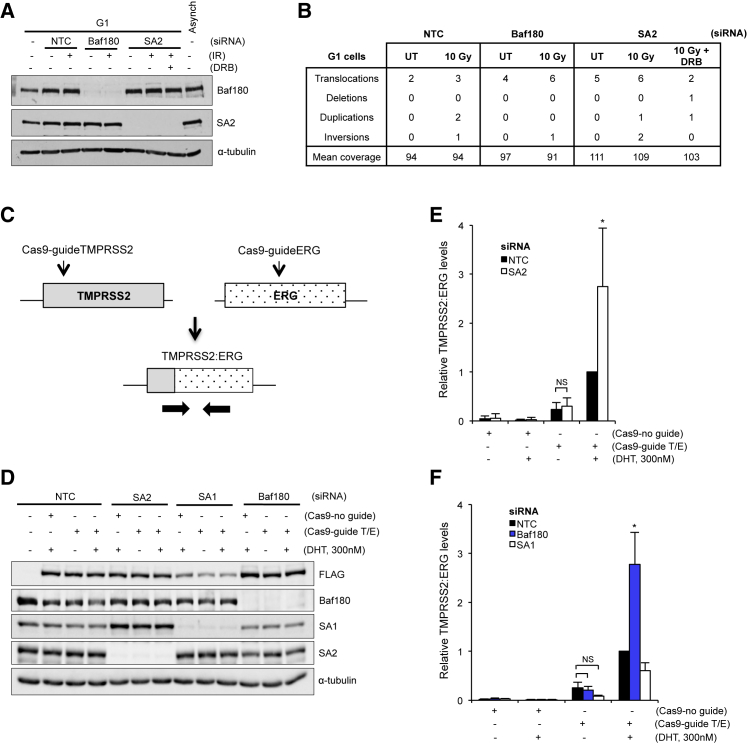
PBAF and Cohesin Are Important for Preventing Chromosome Rearrangements at DSBs in G1, Specifically at DSBs near Strong Transcriptional Activity (A) Western blot analysis of cell extracts prepared from G1-arrested U2OS cells. Cells were depleted of the indicated factors (NTC, non-targeting control) and harvested 6 hr after irradiation with 0 or 10 Gy. DRB was used for 1 hr prior to irradiation in the SA2-depleted cells to inhibit transcription. α-Tubulin was used as a loading control. (B) Table of large-scale genome rearrangements identified in BAF180- or SA2-depleted G1 phase cells treated as in (A) using differential exome sequencing. UT, untreated. DRB was used for 1 hr prior to irradiation in the SA2-depleted cells to inhibit transcription. (C) Schematic illustrating the CRISPR-Cas9 system for generating DNA DSBs in the TMPRSS2 and ERG genes. Guide RNA positions are indicated (Cas9-guideTMPRSS2 and Cas9-guideERG). Translocation between these genes is monitored by qRT-PCR using a forward primer that flanks the fusion and a reverse primer that recognizes the ERG gene. (D) Western blot analysis of whole-cell extracts prepared from LNCaP cells transfected with the indicated siRNAs and FLAG-tagged Cas9 with or without the TMPRSS2 and ERG guide RNAs (Cas9-guideT/E or Cas9-no guide) in the presence or absence of 300 nM DHT. (E and F) Relative TMPRSS2:ERG translocation frequency monitored by qRT-PCR as outlined in (C) in cells treated as in (D). Cells were treated with siRNA targeting SA2 (E), or BAF180 or SA1 (F). NTC, non-targeting control. Data are presented as the mean ± SD; n = 6 (E) n = 3 (F) biological repeats. ^∗^p < 0.05, ^∗∗^p < 0.01 using unpaired Student’s t test. NS, not significant. See also Figure S6.

To rule out known sister chromatid cohesion-dependent repair functions, we monitored misrepair events following depletion of SA2 or BAF180 in irradiated cells held in G1 phase, in which no sister chromatid is present (Figures 6A, 6B, and S6A–S6E). Cells held in G1 and depleted of SA2 or BAF180 were then analyzed by differential exome sequencing (Figure 6B; Gelot et al., 2016).

We found that control cells had an increased number of large-scale genome rearrangements following irradiation (Figure 6B). Cells depleted of either BAF180 or SA2 similarly had an increased number of large-scale rearrangements both with and without irradiation (Figure 6B). These data suggest that PBAF and cohesin function in the G1 phase of the cell cycle to prevent misrepair of DNA DSBs.

We also treated irradiated SA2-depleted cells with 5,6-Dichlorobenzimidazole 1-β-D-ribofuranoside (DRB) to globally inhibit transcription (Figures S6A and S6B). We found that SA2 depletion under these conditions no longer resulted in an increased number of genome rearrangements in irradiated G1 cells (Figure 6B), suggesting that the role of SA2 in preventing genome instability in G1 is related to ongoing transcription.

We wanted to further investigate whether this role in preventing large-scale genome rearrangements is related to repressing transcription at DNA DSBs. To do this, we used a modified protocol to measure translocations between the TMPRSS2 and ERG genes in which the DSBs are introduced at the translocation breakpoints using CRISPR-Cas9 (Li et al., 2018; Figure 6C). This way, DSB induction is no longer dependent on DHT-induced transcription, allowing us to monitor translocation frequency under conditions of different transcriptional activity levels.

We established that DHT treatment did not alter Cas9 expression (Figure S6F) and then monitored translocations using qRT-PCR (Figure 6C). In control cells, introduction of Cas9 together with the guide RNAs resulted in TMPRSS2:ERG rearrangements, and the frequency was increased when the cells were treated with DHT to induce TMPRSS2 transcription (Figure 6E), consistent with the idea that actively transcribed genes are particularly vulnerable to misrepair of DNA DSBs, leading to large-scale rearrangements.

Depletion of SA2 resulted in an increase in translocations, but only when cells were treated with DHT (Figures 6D and 6E), suggesting that it is preventing misrepair only under conditions of strong transcriptional activity. We found that depletion of BAF180 similarly led to an increase in translocations in DHT-treated cells, but not in untreated cells (Figures 6D and 6F). In contrast, there was no increase under any conditions when SA1 was depleted (Figures 6D and 6F). A small decrease in translocations was apparent in these samples, but this may have been due to lower levels of Cas9 expression when SA1 was depleted (Figure 6D).

Together, these data are in line with the idea that SA2 and BAF180 repress transcription near DNA DSBs to promote their accurate repair, and this happens throughout the cell cycle.

### SA2-Deficient Cancers Show Distinct Patterns of Genome Instability

Our data raise the possibility that SA2 (and the cohesin and PBAF complexes) may contribute to preventing tumorigenesis at least in part through their role in DSB-induced transcriptional repression. We therefore investigated whether there was any evidence for loss of this pathway in cancers lacking SA2. To look at this, we analyzed sequencing data from bladder cancer, where there are substantial numbers of available samples with and without mutations in the SA2-encoding gene.

**Figure 7:**
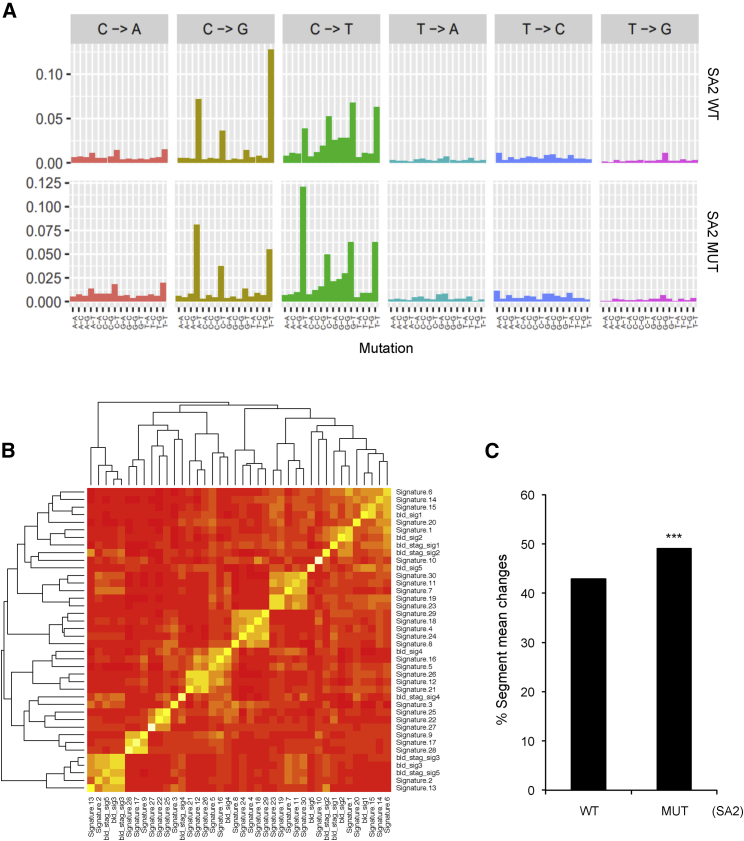
SA2-Deficient Bladder Cancer Samples Show Patterns of Genome Instability that Are Consistent with Loss of Cohesin-Mediated Repair Functions (A) Mutational spectra of all base substitutions observed in SA2-wild-type (top) and SA2 mutant (bottom) bladder cancer samples. Five signatures were identified in each group. See Table S1 for details. (B) Heatmap showing the correlation between the mutational signatures identified in the SA2-stratified bladder cancer samples and those published in COSMIC (Alexandrov et al., 2015). The SA2 mutant cancers (but not the wild-type [WT] bladder cancer samples) had a signature matching COSMIC signature 3, which is annotated as a loss of homologous recombination. (C) Copy-number variation measured as a percentage of segment mean changes present in SA2 mutant or wild-type bladder cancer samples. The SA2 mutant samples had an average of 482 genes with a gain or loss due to copy-number changes, whereas the samples without SA2 mutation had an average of 310. The null hypothesis was that the mutation status of SA2 was independent of the number of genes that had a changed segment mean. ^∗∗∗^p < 0.001 using chi-squared test. See also Table S1.

By performing non-negative matrix factorization (NNMF) of the sequences (as in Polak et al., 2017), we identified 5 signatures associated with SA2-proficient cancers and 5 signatures associated with SA2-deficient cancers (Figure 7A; Table S1). When compared with the existing mutational signatures defined by Nik-Zainal and colleagues (Alexandrov et al., 2015, Nik-Zainal et al., 2012), these signatures clustered into 6 clusters, of which 3 overlapped (Figure 7B; Table S1). These overlapping signatures included signature 1, which is found in all cancers, and signatures 2 and 13, which are thought to be related to the activity of the AID/APOBEC cytidine deaminases and are frequently seen in bladder cancer. Signature 10 was also evident in both groups. The signatures unique to the bladder cancers without SA2 mutations are 15, which is associated with defective mismatch repair, and 16, which has an unknown etiology. The signature that is unique to the SA2-deficient cancers is signature 3 (Table S1), which is associated with defective homologous recombination (HR). Thus, we show that loss of cohesin leads to defective HR, as predicted from its known role in this pathway.

Based on our experimental data, we would predict that the absence of DNA DSB-induced transcriptional repression should additionally lead to increased large-scale rearrangements. Unfortunately, there is little available large-scale chromosomal rearrangement data for bladder cancer. However, copy-number variation (CNV) data are available, and CNV can arise through pathways that include large-scale mis-rejoining events and genome rearrangements (Hastings et al., 2009).

We therefore looked at the changes in copy number using segment mean data in order to interrogate large-scale genome rearrangements, such as duplications and large deletions. We find that there are significantly more segment mean changes in SA2-deficient cancers than in SA2-WT (wild-type) cancers (Figure 7C), consistent with the data we generated in our experimental systems (Figures 5 and 6).

Together with the analysis of the cancer-associated S202L mutation of SA2, which is proficient for sister chromatid cohesion, but not transcriptional repression at DNA breaks (Figure 4), these data raise the possibility that one mechanism by which SA2 functions as a tumor suppressor gene is through its role in promoting accurate repair at DNA DSBs that occur in the vicinity of actively transcribed genes.

## Discussion

Here, we identify a new function for the cohesin complex. We find the centromere-specific cohesin complex subunits, including SA2, are required for repressing transcription near to DNA breaks. While our data suggest that cohesion establishment factors contribute to this activity, we find that this takes place in both G1 and G2 phases of the cell cycle and is therefore not dependent on the presence of a sister chromatid. This is a distinct function from its known roles in promoting repair by HR and by using sister chromatid cohesion to promote accurate NHEJ (Gelot et al., 2016).

We find that failure to repress transcription near DNA DSBs can lead to large-scale genome rearrangements, such as translocations. One mechanism by which repressing transcriptional activity could help to prevent these rearrangements is by providing unobstructed access to the repair machinery. The RNA polymerase may disrupt tethering of the broken DNA ends, leading to an increased possibility of misrepair. In addition, since actively transcribed genes are preferentially repaired by HR (at least in S and G2 phase cells; Aymard et al., 2014), the transcription machinery could impede resection and/or strand invasion, which require long stretches of chromatin. While we find a role for this pathway throughout interphase, it is possible that the consequences of its loss are greater in S and G2 phases, when HR is available.

We show that cohesin loading and establishment factors are required for repressing transcription near DNA breaks, and this is true in G1 and G2 phase cells. Cohesin may be looping the DNA in areas proximal to the break in order to reorganize the chromosome region. This could not only directly impact transcription but also remove the broken DNA end from the vicinity of other actively transcribed genes to prevent misrepair.

Our data suggest that SA2, but not SA1, is required for repressing transcription near DNA breaks. Recently, SA2 has been shown to bind to DNA structures such as double-stranded DNA ends, single-stranded overhangs, flaps, and single-stranded DNA (ssDNA) gaps (Countryman et al., 2018), suggesting one mechanism by which it could specifically function in this pathway. In addition, it was recently found that SA2 and SA1 play distinct roles in genome organization (Kojic et al., 2018). Specifically, SA2 appears to associate more dynamically with chromatin and has a greater role in mediating gene expression than SA1, which contributes more to topological organization of the genome (Kojic et al., 2018). These properties of SA2 make it well suited to be employed as part of the DNA damage response, associate with damaged chromatin, and regulate the expression of nearby genes.

Cohesin plays multiple cellular roles, many of which can impact on genome stability and cellular identity. Consequently, the mechanisms by which the cohesin complex acts to prevent tumorigenesis are an outstanding question in the field (Hill et al., 2016). While defective sister chromatid cohesion leading to aneuploidy could drive cancer progression, it is clear that this is not the mechanistic explanation in all cases. There is also evidence that misregulation of transcription in the absence of cohesin plays a role in at least some cancers (Mazumdar et al., 2015). In addition, cohesin makes multiple contributions to DNA repair (Brough et al., 2012, Gelot et al., 2016, Kong et al., 2014), and our analysis of mutational signatures associated with loss of SA2 (Figure 7) suggests that HR deficiency may be a contributing factor to tumorigenesis in these cells. Here, we provide evidence that transcriptional repression in response to nearby DNA DSBs also contributes to genome stability. This function is an important component of cohesin’s suite of functions that may contribute to its role in preventing tumorigenesis.

## STAR★Methods

### Key Resources Table

**Table:** 

REAGENT or RESOURCE	SOURCE	IDENTIFIER
**Antibodies**		

Mouse monoclonal anti-ARID2 (E-3)	Santa Cruz	sc-166117; RRID: AB_2060382
Rabbit monoclonal anti-ATM (D2E2)	Cell Signaling Technology	2873; RRID: AB_2062659
Rabbit polyclonal anti-Baf180	Millipore	ABE70; RRID: AB_10807561
Rabbit polyclonal anti-BMI1	Bethyl Labs	A301-694A; RRID: AB_1210891
Mouse monoclonal anti-BRG1 (G7)	Santa Cruz	sc-17796; RRID: AB_626762
Rabbit polyclonal anti-CENPF	Bethyl Labs	A301-611A; RRID: AB_1210906
Rabbit polyclonal anti-CTCF	Millipore	07-729; RRID: AB_441965
Rabbit polyclonal anti-Cyclin D1	Neomarkers	RB-010-PO
Rabbit polyclonal anti-Esco2	Abcam	ab86003; RRID: AB_1924967
Rabbit monoclonal anti-EZH2	Cell Signaling Technology	5246; RRID: AB_10694683
Mouse monoclonal anti-GFP (B2)	Santa Cruz	sc-9996; RRID: AB_627695
Mouse monoclonal anti-H2A-K119ub (E5C5)	Millipore	05-678; RRID: AB_309899
Rat monoclonal anti-NIPBL	Abcam	ab106768; RRID: AB_10859516
Rabbit polyclonal anti-PDS5A	Abcam	ab122352; RRID: AB_11129705
Rabbit polyclonal anti-PDS5B	Abcam	ab70298; RRID: AB_1269710
Rabbit polyclonal anti-Rad21	Abcam	ab992; RRID: AB_2176601
Rabbit polyclonal anti-SMC3	Abcam	ab155587
Goat polyclonal anti-SA1	Abcam	ab4457; RRID: AB_2286589
Goat polyclonal anti-SA2	Abcam	ab4463; RRID: AB_304471
Rabbit polyclonal anti-WAPL	Abcam	ab70741; RRID: AB_2216719
Mouse monoclonal anti-α-tubulin	Abcam	ab7291; RRID: AB_2241126
Mouse monoclonal anti-γ-H2AX (pSer139) (JBW301)	Millipore	05-636; RRID: AB_309864
Goat Anti-Rabbit Immunoglobulins/HRP	Agilent (Dako)	P044801-2
Rabbit Anti-Mouse Immunoglobulins/HRP	Agilent (Dako)	P026002-2; RRID: AB_2636929
Rabbit Anti-Goat Immunoglobulins/HRP	Agilent (Dako)	P044901-2
Goat Anti-Rat Immunoglobulins/HRP	Millipore	AP136P; RRID: AB_91300
Goat Anti-Mouse IgG/FITC	Sigma-Aldrich	F0257-1ML
Sheep Anti-rabbit IgG/Cy3	Sigma-Aldrich	C2306-1ML
Goat Anti-Mouse IgG/Alexa Fluor 555	Thermo Fisher Scientific (Invitrogen)	A-21422; RRID: AB_141822

**Chemicals, Peptides, and Recombinant Proteins**		

4-hydroxytamoxifen	Sigma-Aldrich	H7904-5MG
5,6-Dichlorobenzimidazole 1-β-D-ribofuranoside (DRB)	Sigma-Aldrich	D1916-10MG
ATM Kinase Inhibitor	Santa Cruz Biotechnology	sc-202963 (CAS 587871-26-9)
cOmplete, Mini, EDTA-free Protease Inhibitor Cocktail	Sigma-Aldrich (Roche Applied Science)	04693159001
5α-Dihydrotestosterone (DHT)	Sigma-Aldrich	D-073-1ML
Doxycycline hyclate	Sigma-Aldrich	D9891-G
Hoechst 33258 solution	Sigma-Aldrich	94403-1ML
Propidium iodide	Thermo Fisher Scientific (Invitrogen)	P3566
RNase A	Sigma-Aldrich	R5503-100MG
Sheild1	Clontech Laboratories UK Ltd	632189
Thymidine	Sigma-Aldrich	T1895-5G

**Critical Commercial Assays**		

Click-iT RNA Alexa Fluor 594 Imaging Kit	Thermo Fisher Scientific (Invitrogen)	C10330
HiPerFect Transfection Reagent	QIAGEN	301705
Lipofectamine RNAiMAX Transfection Reagent	Thermo Fisher Scientific (Invitrogen)	13778150
Lipofectamine LTX Reagent with PLUS Reagent	Thermo Fisher Scientific (Invitrogen)	15338100
Nucleospin Tissue	Machery-Nagel	740952.5
Power SYBR green PCR master mix	Thermo Fisher Scientific (Applied Biosystems)	4367659
Premo FUCCI Cell Cycle Sensor (BacMam 2.0)	Thermo Fisher Scientific (Invitrogen)	P36237
QIAGEN RNeasy Mini Kit	QIAGEN	74106
QuikChange II Site-Directed Mutagenesis Kit	Agilent	200523
SuperScript First-Strand Synthesis System for RT-PCR	Thermo Fisher Scientific (Invitrogen)	11904018
TurboFect Transfection Reagent	Thermo Fisher Scientific	R0531
TMPRSS2/ERG Deletion/Breakapart Probe	Cytocell	LPS 021

**Deposited Data**		

Differential exome sequencing data and files	This paper	SAMN08226046
Human reference genome NCBI build 37, GRCh37	Genome Reference Consortium	https://www.ncbi.nlm.nih.gov/projects/genome/assembly/grc/human/
Genomic data commons	Grossman, 2016	N/A
COSMIC database	Forbes et al., 2017	https://cancer.sanger.ac.uk
Raw data files deposited in Mendeley Data	This paper	https://doi.org/10.17632/4h486ty62f.1

**Experimental Models: Cell Lines**		

U2OS	From cell line stocks at GDSC, Sussex University, validated by STR profiling with ECACC	N/A
U2OS 263 IFII	Gift from Roger Greenberg; Tang et al., 2013	N/A
U2OS 265	Gift from Roger Greenberg; Tang et al., 2013	N/A
LNCaP clone FGC	ATCC	CRL-1740
U2OS Baf180 KO CRISPR Clone 15	This paper	N/A
U2OS NTC sh	Hopkins et al., 2016	N/A
U2OS Baf180 sh	Hopkins et al., 2016	N/A

**Oligonucleotides**		

siRNA sequence: ARID2 SMARTpool: ON-TARGETplus	Dharmacon	L-026945-01-0005
siRNA sequence: Baf180 SMARTpool: ON-TARGETplus	Dharmacon	L-008692-01-0005
siRNA sequence: BRG1 SMARTpool: ON-TARGETplus	Dharmacon	L-010431-00-0005
siRNA sequence: BRM SMARTpool: ON-TARGETplus	Dharmacon	L-017253-00-0005
siRNA sequence: CTCF SMARTpool: ON-TARGETplus	Dharmacon	L-020165-00-0005
siRNA sequence: Esco2 SMARTpool: ON-TARGETplus	Dharmacon	L-025788-01-0005
siRNA sequence: NIPBL SMARTpool: ON-TARGETplus	Dharmacon	L-012980-00-0005
siRNA sequence: NTC: Non-targeting pool: ON-TARGETplus	Dharmacon	D-001810-10-20
siRNA sequence: Rad21 SMARTpool: ON-TARGETplus	Dharmacon	L-006832-00-0005
siRNA sequence: SMC3 SMARTpool: ON-TARGETplus	Dharmacon	L-006834-00-0005
siRNA sequences for ATM, NTC, PDS5A, PDS5B, Sororin, STAG1, STAG2, WAPL, see Table S4	This paper	N/A
Primer: TMPRSS2_rtPCR FWD: CTGGTGGCTGATAGGGGAT	Lin et al., 2009	N/A
Primer: TMPRSS2_rtPCR REV: GTCTGCCCTCATTTGTCGAT	Lin et al., 2009	N/A
Primer: TMPRSS2-CR-3F: CACCGTTCATTCACGATCCCTAACA	Li et al., 2018	N/A
Primer: TMPRSS2-CR-3R: AAACTGTTAGGGATCGTGAATGAAC	Li et al., 2018	N/A
Primer: ERG-CR-2F: CACCGGGATGGTAAACGGAGAGTGC	Li et al., 2018	N/A
Primer: ERG-CR-2R: AAACGCACTCTCCGTTTACCATCCC	Li et al., 2018	N/A
Primer: TMPRSS2:ERG FWD: AGCGCGGCAGGAAGCCTTAT	Sigma	N/A
Primer: TMPRSS2:ERG REV: CCGTAGGCACACTCAAACAACGA	Mani, 2016	N/A
Primer: Reporter Transcript FWD: TCATTAGATCCTGAGAACTTCA	Shanbhag et al., 2010	N/A
Primer: Reporter Transcript REV: TTTTGGCAGAGGGAAAAAGA	Shanbhag et al., 2010	N/A
Primer: Actin_rtPCR FWD: GCTCGTCGTCGACAACGGCTC	Lin et al., 2009	N/A
Primer: Actin_rtPCR REV: CAAACATGATCTGGGTCATCTTCTC	Lin et al., 2009	N/A
Primer: cyclophilin A_rtPCR FWD: CTGGACCCAACACAAATGGT	This paper	N/A
Primer: cyclophilin A_rtPCR REV: GCCTTCTTTCACTTTGCCAAAC	This paper	N/A
Primer: Sororin_rtPCR FWD: AGTCTCGCCAGTGGTGTGCT	Zhang, 2011	N/A
Primer: Sororin_rtPCR REV: TTCAACCAGGAGATCAAACTGC	Zhang, 2011	N/A
Primer: GAPDH_rtPCR FWD: ACATCGCTCAGACACCATG	Meisenberg, 2015	N/A
Primer: GAPDH_rtPCR REV: TGTAGTTGAGGTCAATGAAGGG	Meisenberg, 2015	N/A
Primers for Site Directed Mutagenesis and creation of Rad21-pEGFP-C1 and STAG2-pmRFP-C1, see Table S4	This paper	N/A

**Recombinant DNA**		

Plasmid: Baf180-pEGFP-C3	Kakarougkas et al., 2014	N/A
Plasmid: STAG2-pEGFP-C1	Solomon, 2011	Addgene plasmid: 31972
Plasmid: STAG2-pmRFP-C1	This paper	N/A
Plasmid: Rad21-pEGFP-C1	This paper	N/A
Plasmid: pEGFP-C1	Gift from Keith Caldecott (University of Sussex)	N/A
Plasmid: pmRFP-C1	Gift from Keith Caldecott (University of Sussex)	N/A
Plasmid: Cas9-gRNA	Horizon	free CRISPR guide program
pcDNA4-GFP-IRES-Puro	Gift from Keith Caldecott (University of Sussex)	N/A
pSpCas9(BB)-2A-Puro (PX459) V2.0	Gift from Helfrid Hochegger (University of Sussex)	N/A

### Contact for Reagent and Resource Sharing

Further information and requests for resources and reagents should be directed to and will be fulfilled by the Lead Contact, Jessica A. Downs (Jessica.Downs@icr.ac.uk).

### Experimental Model and Subject Details

#### Cell Lines and Cell Culture

The U2OS, U2OS Baf180 KO and Baf180sh, and U2OS reporter cell lines (U2OS 263 IFII and U2OS 265, Tang et al., 2013) were maintained in a 5% CO_2_ incubator at 37°C in GIBCO DMEM media (Life Technologies, Paisley, UK) supplemented with 100 U/mL penicillin/streptomycin, 2 mM L-glutamine and 10% FCS (U2OS) or 10% TET System approved FCS (U2OS reporter cell lines; 631106, Takara Bio).

LNCaP cells were obtained from ATCC (Clone FGC, CRL-1740) and cultured in GIBCO RPMI media (Life Technologies, Paisley, UK) supplemented with 10% FCS, 2 mM L-glutamine, 100 U/mL penicillin/streptomycin, 10 mM HEPES and 1 mM sodium pyruvate. All cell lines were regularly tested for mycoplasma contamination.

### Method Details

#### CRISPR-Cas9 U2OS PBRM1 (BAF180) KO

Guide RNA sequences were integrated within an all-in-one Cas9-gRNA vector (Horizon – free CRISPR guide program). A second plasmid to aid cell selection was pcDNA4-GFP-IRES-Puro, a gift from Prof. Keith Caldecott (University of Sussex, UK). The genomic sequence targeted for CRISPR-Cas9 disruption in *PBRM1* was ATAGAAGAAGTTGGATTCCA. U2OS were co-transfected in a ratio of 2:1 (Cas9-gRNA: pcDNA4-GFP-IRES-Puro) using TurboFect (R0531, Thermo Fisher Scientific). Transgene expression was analyzed 24 hr after transfection and cells were put under 1.5 μg/ml puromycin selection for at least 72 hr. Single cells were isolated and grown for approximately three weeks. Genomic DNA was isolated, and successful clones were determined by SURVEYOR mutation detection assay (706020, Integrated DNA technologies). Clones were expanded in culture and *PBRM1* knockouts were identified by western blotting using a Baf180 antibody and sequencing (GATC-biotech).

#### siRNA-Mediated Depletion and Expression Construct Transfection

LNCaP cells were transfected with the indicated siRNA in two rounds separated by 24 hr using Lipofectamine RNAiMAX transfection reagent (13778150, Invitrogen, Paisley, UK) as per manufacturer’s guidelines. The U2OS cell lines were transfected with the indicated siRNA in two rounds (24 hr apart) on cells in suspension using HiPerfect (301705, QIAGEN, Crawley, UK). Briefly, 12 μL HiPerfect reagent added to 200 μL Optimem (31985062, GIBCO, Life Technologies, Paisley, UK) was incubated at room temperature for 5 min prior to the addition of siRNA oligonucleotides. The mixture was incubated for 20 min and added to 3.5x10^5^ U2OS cells suspended in 4 mL media (6 cm dish). Cells were analyzed 72 hr following second transfection. siRNA sources and sequences are outlined in Table S3.

#### Transfection Protocol

Plasmid transfection was carried out using Lipofectamine LTX and Plus reagents (15338100, Invitrogen, Paisley, UK) as per manufacturer’s guidelines. For micro-irradiation tracking, 6-8 μL LTX reagent and 1-2.5 μL Plus reagent was used to transfect 2-4 μg plasmid on to 1 × 10^5^ adhered U2OS cells in 2 mL media in a 3.5 cm glass bottom dish (P35G-1.5-14-C, MATEK Corporation). For the U2OS transcription reporter assay, 8 μL LTX reagent and 2.5 μL Plus reagent was used to transfect 4 μg siRNA resistant pmRFP-C1-STAG2 plasmids into 3.5 × 10^5^ adhered U2OS 263 IFII cells plated in 4 mL media in a 6 cm dish. The Premo FUCCI Cell Cycle Sensor BacMam 2.0 system (15 μL; P36237, ThermoFisher Scientific) was used to infect 1 × 10^5^ adhered U2OS cells plated in 2 mL media in a 3.5 cm glass bottom dish, 16 hr prior to plasmid transfection.

#### Plasmids

pEGFP-C3-Baf180 was previously described in Kakarougkas et al. (2014) and pEGFP-STAG2-wild-type was obtained from Addgene (plasmid # 31972; Solomon et al., 2013). STAG2 cDNA was amplified from the pEGFP-STAG2-wild-type plasmid and inserted between *Hind*III and *Kpn*1 of the pmRFP empty vector. Rad21 cDNA was generated from extracted U2OS mRNA using the Superscript II First Strand RT-PCR system (11904018, Invitrogen, Paisley, UK) according to the manufacturer’s protocol. cDNA was amplified and inserted between *Hind*III and SalI of the pEGFP-C1 empty vector. Both pmRFP-C1 and pEGFP-C1 empty vectors were a gift from Keith Caldecott (University of Sussex). Primers used are outlined in Tables S4.

#### Site-Directed Mutagenesis

Site-directed mutagenesis was carried out using QuikChange II Site-Directed Mutagenesis Kit (200523, Agilent) as per manufacturer’s guidelines. For the pmRFP-C1-STAG2 plasmid, siRNA resistance to the STAG2-08 sequence was generated by introducing four silent mutations using 2 sequential primer sets, followed by site-directed mutagenesis to create the STAG2 S202L or V181M mutations. Selected clones were validated by sequencing. Primers used are outlined in Table S4.

#### U2OS Transcription Reporter Assay

The U2OS 263 IFII transcription reporter cells (Tang et al., 2013) with siRNA transfection seeded on to coverslips were treated with 1 μM Sheild1 (632189, Clontech Laboratories UK Ltd) and 1 μM 4-hydroxytamoxifen (4-OHT) (H7904-5MG, Sigma-Aldrich) for 3 hr to induce mCherry-FokI expression and 1 μg/ml doxycycline hyclate (D9891-G, Sigma-Aldrich) for an additional 3 hr to induce reporter gene transcription. For samples treated with ATM kinase inhibitor, 10 μM inhibitor (CAS 587871-26-9; sc-202963, Santa Cruz Biotechnology) was added 1 hr prior to doxycycline hyclate addition. Cells on coverslip were fixed in 4% paraformaldehyde (15714-5, Electron Microscopy Science), permeabilised for 3 min in 0.2% Triton-X/PBS, washed and mounted on to slides using VECTASHIELD Antifade Mounting Medium with DAPI (H-1200, Vector Laboratories). Cells were visualized using a Nikon Eclipse e-400 microscope with 60X oil objective and the number of transcription positive cells were counted from a total of 150 cells per variable in each independent repeat. For G1/G2 analysis, fixed cells were subject to CENPF (G2 marker) and Cyclin D1 (G1 marker) immunostaining (as below) using a Cy3 labeled secondary antibody. Slides were subsequently imaged on an Olympus IX73 microscope fitted with a Hamatsu Ocra-Flash 4.0 CMOS camera using the Micromanager ImageJ plugin and a 40X oil objective. The number of transcription positive cells scored manually from images for n > 100 CENPF or Cyclin D1 positive cells per variable in each independent repeat.

#### EU Incorporation Assay

The Invitrogen Click-iT RNA Alexa Fluor 594 Imaging Kit (C10330, ThermoFisher Scientific) was used to measure RNA synthesis at laser micro-irradiated sites in the U2OS cell line. Briefly, 1 × 10^5^ siRNA treated U2OS cells were transferred to 3.5 cm glass bottom dishes (P35G-1.5-14-C, MatTek Corporation) at the second siRNA hit in suspension as described above. Three days later, the adhered cells were laser microirradiated with a 405 nm UV-laser at a dose of 0.175 μJ/μm^2^ following a 30 min incubation in media containing 10 μg/mL Hoechst 33258 (94403-1ML, Sigma-Aldrich) as described below. Within 10 min of microirradiation, the media was replaced with media containing the EU component at recommended concentration. After a 45 min incubation, cells were fixed and the EU Click-IT assay carried out as per manufacturer’s guidelines. Before mounting, the cells were immunostained for γH2AX as described below using FITC-labeled anti-mouse secondary antibodies. The coverslips were separated from the MATEK dishes and mounted on to slides using Vectashield mounting media containing DAPI prior to imaging. The micro-irradiated cells were identified by γH2AX signal for single plane imaging using a 100X oil objective on a Zeiss microscope fitted with a Hamamatsu Orca ER camera and Micromanager ImageJ software. ImageJ software was used to analyze the EU intensity profile across the damaged region (γH2AX positive) using a line tool (69 pixels in length and 15 pixels in width) placed at a 90**°** angle to damage stripe, centered at the γH2AX positive region with ends at non-damaged regions. Regions of intense staining (likely corresponding to nucleoli) were avoided. The EU intensity reads across the line were normalized to each end, and averaged to create the intensity plots. At least 30 cells were analyzed per variable in each independent repeat.

#### Whole-Cell Extract Preparation and Western Blotting

Cell pellets were lysed for 30 min in ice-cold lysis buffer (20 mM Tris-HCl pH 7.5, 10 mM EDTA pH 8.0, 100 mM NaCl, 1% Triton X-100) supplemented with Complete Mini EDTA-free Protease Inhibitor Cocktail (04693159001, Roche Applied Science, Burgess Hill, UK) and sonicated for 2 cycles 30sON-30sOFF at 4°C. The lysate was cleared by centrifugation at 13,000 rpm for 10 min and supernatant collected for protein measurement by Bradford assay and storage at −80°C. For western blotting, WCE (40 μg) was separated by 8% SDS-polyacrylamide gel electrophoresis (PAGE) for 2 hr at 125 V followed by transfer onto a Hybond-C Extra Nitrocellulose membrane (Fisher Scientific UK, Loughborough, UK) for 90 min at 30 V. The membrane was blocked in 5% PBS-milk for 1 hr and probed overnight with diluted primary antibodies. The membrane was thrice washed in 0.1% TBS-Tween-20, incubated with secondary antibodies diluted in 5% PBST-milk for 1 hr, washed three times in 0.1% TBS-Tween-20 prior to film development using in-house ECL reagent. See Table S2 for antibody and dilution information.

#### γH2AX Foci Analysis

Following siRNA depletion as described above, U2OS cells adhered on coverslips were exposed to 1.5 Gy caesium-137 γ-irradiation (GammaCell 1000, Atomic Energy of Canada Ltd). At indicated time points, cells were analyzed by immunostaining with an antibody against γH2AX and FITC-labeled secondary as described below. For G1 analysis, cells were also immunostained with Cyclin D1 and a Cy3-labeled secondary. Cells were visualized using a Nikon Eclipse e-400 microscope with 60X objective and the number of γH2AX foci per cell, or per Cyclin D1-positive (G1) cell, was counted from a total of 36 cells per variable in each independent repeat.

#### Immunostaining

Sub-confluent cells on coverslips were washed twice in PBS prior to fixing with 4% paraformaldehyde for 20 min, the washed three times in 1xPBS and permeabilised in 0.2% Triton-X/PBS for 3 min. Coverslips were washed, blocked for 20 min in 2% BSA-Fraction V (A3059-50G, Sigma-Aldrich), and followed by a 1 hr incubation with primary antibody diluted in 2% BSA-Fraction V. The coverslips were washed three times with 1xPBS, then incubated with labeled secondary antibodies diluted 1:300 in 2% BSA-Fraction V for 45 min. Coverslips were wash three times in 1xPBS, mounted on to slides using DAPI Vectashield (H-1200, Vector Laboratories) and stored at 4°C for further analysis. See Table S2 for antibody and dilution information.

#### Laser Micro-irradiation

For laser tracking micro-irradiation experiments, 1.5 × 10^5^ U2OS cells seeded in 3.5 cm glass bottom dishes (P35G-1.5-14-C, MatTek Corporation) were transfected with FUCCI cell cycle markers 44h prior to micro-irradiation and expression plasmids 28h prior to microirradiation as described above. Cells were subject to a 30 min incubation in media containing 10 μg/mL Hoechst 33258 (94403-1ML, Sigma-Aldrich) before micro-irradiating with a 405nm UV laser at a dose of 0.452 μJ/μm^2^ using a 60X oil objective on an Olympus 3I Spinning Disk microscope. Micro-irradiation was set to trigger at the second time point and the integrated EMCCD Evolve camera captured time-lapse images at 10 s intervals for 5 min per cell. A minimum of 20 cells per variable was analyzed per independent repeat. Quantification was carried out using Spinning Disk Slidebook software.

#### QRT-PCR Analysis of TMPRSS2 Expression

Prior to 5α-Dihydrotestosterone (DHT) and irradiation (IR) treatment, LNCaP cells were washed twice in serum free RPMI and cultured for 72 hr in complete RPMI supplemented with 5% charcoal stripped serum instead of 10% FCS. Cells were then irradiated with 10 Gy caesium-137 γ-irradiation (GammaCell 1000, Atomic Energy of Canada Ltd) and treated with 300 nM DHT (D-073-1ML, Sigma-Aldrich) for the times indicated in each figure. RNA was extracted from 1 × 10^6^ LNCaP cells using RNAeasy Mini Kit (74106, QIAGEN) according to the manufacturer’s protocol. cDNA was synthesized from 1 μg RNA using Superscript II First Strand RT-PCR system (11904018, ThermoFisher Scientific) according to the manufacturer’s protocol. Quantitative PCR was performed using a StepOnePlus Real-Time PCR System (Applied Biosystems) on reactions prepared with Power SYBR green PCR master mix (4367659, ThermoFisher Scientific) using 2.5% (25 ng) of the cDNA for TMPRSS2 expression analysis. The cyclophilin A gene was used for normalization. Primers are listed in Table S4.

#### Assay for IR-Induced TMPRSS2:ERG Translocations by FISH or Quantitative PCR

Prior to 5α-Dihydrotestosterone (DHT) and irradiation (IR) treatment, LNCaP cells were washed twice in serum free RPMI and cultured for 72 hr in complete RPMI supplemented with 5% charcoal stripped serum instead of 10% FCS. LNCaP cells were harvested with trypsin 24 hr after addition of 300 nM DHT (D-073-1ML, Sigma-Aldrich) and treatment with 10 Gy using caesium-137 γ-irradiation (GammaCell 1000, Atomic Energy of Canada Ltd). For quantitative PCR, mRNA and cDNA were prepared as above and qPCR carried out using the TMPRSS2:ERG primer set. For FISH analysis, cells were washed in PBS and fixed for 15 min with Carnoy’s Fixative (3:1 methanol:acetic acid) that was added dropwise to cells while gently vortexing. The suspension was centrifuged at 1500 RPM for 8 min, the fixation step repeated and the cells resuspended in 30 μL Carnoy’s fixative. Cells were dropped onto a microscope slide and dried. The slide was rehydrated in 2X SSC (20X SSC: 3 M NaCl, 300 mM trisodium citrate, pH 7.0) for 2 min followed by dehydration in an ethanol series (70, 80 and 95%) for 2 min each at room temperature. The TMPRSS2/ERG Deletion/Breakapart Probe mix (LPS 021, Cytocell) and slide were separately incubated for 5 min at 37°C before addition of the probe mix to the slide, addition of a coverslip and sealing with rubber glue. The slide was heated at 75°C for 5 min before overnight incubation at 37°C. The coverslip was removed, the slide washed in 0.4X SSC for 2 min at 72°C and then in 2X SSC with 0.05% Tween-20 for 30 s at room temperature. The coverslip was mounted on slides using DAPI and imaged with a 40X objective on an Olympus IX71 microscope fitted with a CoolSNAP HQ2 camera and Micromanager ImageJ software. A total of 50 imaged cells per variable were scored manually as unrearranged or rearranged (example images in Figure 5E).

#### Assay for CRISPR-Cas9-Induced TMPRSS2:ERG Translocations

CRISPR guide RNA sequences against the TMPRSS2 gene (Primer pair TMPRSS2-CR-3) and the ERG gene (Primer pair ERG-CR-2) (from Li et al., 2018) were cloned in to the Cas9-empty plasmid (pSpCas9(BB)-2A-Puro (PX459) V2.0 plasmid (Ran, 2013) using BbsI-HF (NEB; R3539S) restriction enzyme to create Cas9-TMPRSS2 and Cas9-ERG plasmids. LNCaP cells subjected to the 2-hit siRNA depletion in CSS-RPMI media were trypsinised on day 3, counted and 4x10ˆ5 cells transfected with either 6μg Cas9-empty plasmid or 3μg each for plasmids Cas9-TMPRSS2 and Cas9-ERG in CSS RPMI media with or without 300nM DHT. The media was replaced with or without 300nM DHT 24hr later and cells harvested at 72hr for analysis by QRT-PCR using TMPRSS2-ERG translocation primers, as above.

#### Cell-Cycle Synchronization and FACS Analysis

siRNA treated U2OS cells were synchronized in G1 phase using a double thymidine block. Specifically, 7.5 × 10^5^ siRNA transfected U2OS cells were treated with 2.5 mM thymidine in media for 19 hr. Cells were washed twice with PBS and incubated in media for 9h, prior to the addition of 2.5 mM thymidine for 16 hr to synchronize cells in G1 (this time point is referred to as ‘0 hr’ in figure S6A). For fluorescence-activated cell sorting (FACS) analysis of cell cycle profile, cells were trypsinized, washed twice in ice-cold PBS and resuspended in 0.5 mL 1X PBS. Cells were fixed by gently vortexing while adding 4.5 mL ice-cold 70% ethanol drop-wise followed by 30 min incubation at 4°C. Fixed cells were washed twice in 1X PBS and resuspended in 0.5 mL Staining Solution (0.1% Triton X-100, 10 μg/mL propidium iodide (P3566, ThermoFisher Scientific), 100 μg/mL RNase A (R5503-100MG, Sigma-Aldrich)) and incubated for 30 min in the dark at room temperature. Cells were analyzed on a BD LSR II Flow Cytometer (BD Biosciences) and profiles generated using FlowJo v10.1 software.

#### Exome Sequencing Analysis

U2OS cells were subject to siRNA depletion and double thymidine block to synchronize cells in G1 (as described above). After 24 hr, one set of each siRNA treated sample was irradiated with 10 Gy using caesium-137 γ-irradiation (GammaCell 1000, Atomic Energy of Canada Ltd, Canada). One hour prior to irradiation, one set of the SA2-depleted cells were treated with 75 μM DRB. All samples were collected 6h after irradiation. Genomic DNA was isolated using Nucleospin Tissue kit (740952.5, Machery-Nagel). Exome capture was performed, and the library was subjected to paired-end sequencing using a HiSeq2500 system (Illumina) at the Tumor Profiling Unit (Institute of Cancer Research) to a median depth of 100X per sample.

BWA (version 0.7.5a) was used to align reads to the human reference genome (GRCh37). PCR duplicates were removed prior to further processing and variant detection. Variants were called using Genome Analysis Tool Kit (GATK; version 2.7-2) and MuTect (version 1.1.4) Broad Best Practices Pipeline using standard settings, and the structural rearrangements were identified using Delly2. The U2OS cell line was sequenced in parallel so that changes in the experimental samples that were shared with the parental U2OS sample were removed.

#### Analysis of Cancer Samples

To investigate the difference in mutational patterns in SA2 competent and SA2 deficient tumors, mutational fingerprints for two groups of patients were generated (Polak et al., 2017) using mutational data from whole genome screens annotated in the COSMIC database https://cancer.sanger.ac.uk (Forbes et al., 2017). One group included samples from 336 bladder cancer patients that did not exhibit a SA2 mutation and the other group included samples from 38 bladder cancer patients with a SA2 mutation.

For both groups of samples their mutational fingerprints were decomposed using a non-negative matrix factorisation to produce 5 signatures. Decomposition was performed using the Brunet method (Brunet et al., 2004) through the NMF library in R3.4.0. The resulting signatures were compared to those published in COSMIC using a correlation matrix produced again in R using the Pearson’s correlation method.

To determine whether or not there is a link between large-scale chromosomal alterations and inactivating mutations in SA2, we used copy number variance data. We first explored whether or not there is a link between large-scale chromosomal rearrangements and CNV using COSMIC CNV data for an exome screen on breast cancers where information on chromosomal rearrangements is available. This included 571 samples with documented structural changes and 1175 changes that did not. There were 22-fold more genes affected by CNV changes in samples with structural changes than in samples without (12,505 versus 571). Using a chi squared test, the chance of this happening with independent distributions has a vanishing p value. Therefore, we used CNV data to investigate whether SA2-deficient cancer samples have a greater number of large-scale chromosome rearrangements than SA2-proficient cancer samples. Copy number variance data were available for 409 TCGA bladder cancer samples from the genomic data commons and, as before this was combined with matching somatic mutation data from COSMIC. Copy number variance data were then mapped to gene positions and the segment mean calculated in order to identify for each gene whether or not it had changed significantly. Cutoffs of −0.2 and 0.2 were used in accordance with common practice (Laddha et al., 2014). Our null hypothesis was that there is no correlation between the mutation status of SA2 in a sample and the number of genes that had a changed segment mean. Samples were then split into two groups, 11 where SA2 had a non-synonymous mutation and 398 where they did not. A chi-square test showed that our null hypothesis has a vanishing chance of being correct (p = 0.0).

### Quantification and Statistical Analysis

Statistical details of experiments (numbers of biological replicates and use of standard deviation or standard error) are included in the Figure legends and/or the specific methods section. Significance for Student’s t test or Chi Squared analyses are indicated in figures as ^∗^ = p < 0.05, ^∗∗^ = p < 0.01 and ^∗∗∗^ = p < 0.001.

### Data and Software Availability

Raw data files have been deposited in Mendeley Data (https://doi.org/10.17632/4h486ty62f.1).

The accession number for the exome sequencing data reported in this paper is NCBI SRA: SAMN08226046.

## Supplemental Information

Document S1. Figures S1–S6 and Tables S1–S4

Document S2. Article plus Supplemental Information

## References

[bib1] Alexandrov L.B., Jones P.H., Wedge D.C., Sale J.E., Campbell P.J., Nik-Zainal S., Stratton M.R. (2015). Clock-like mutational processes in human somatic cells. Nat. Genet..

[bib2] Aymard F., Bugler B., Schmidt C.K., Guillou E., Caron P., Briois S., Iacovoni J.S., Daburon V., Miller K.M., Jackson S.P., Legube G. (2014). Transcriptionally active chromatin recruits homologous recombination at DNA double-strand breaks. Nat. Struct. Mol. Biol..

[bib3] Balbás-Martínez C., Sagrera A., Carrillo-de-Santa-Pau E., Earl J., Márquez M., Vazquez M., Lapi E., Castro-Giner F., Beltran S., Bayés M. (2013). Recurrent inactivation of STAG2 in bladder cancer is not associated with aneuploidy. Nat. Genet..

[bib4] Bot C., Pfeiffer A., Giordano F., Manjeera D.E., Dantuma N.P., Ström L. (2017). Independent mechanisms recruit the cohesin loader protein NIPBL to sites of DNA damage. J. Cell Sci..

[bib5] Brough R., Bajrami I., Vatcheva R., Natrajan R., Reis-Filho J.S., Lord C.J., Ashworth A. (2012). APRIN is a cell cycle specific BRCA2-interacting protein required for genome integrity and a predictor of outcome after chemotherapy in breast cancer. EMBO J..

[bib6] Brownlee P.M., Chambers A.L., Cloney R., Bianchi A., Downs J.A. (2014). BAF180 promotes cohesion and prevents genome instability and aneuploidy. Cell Rep..

[bib7] Brunet J.P., Tamayo P., Golub T.R., Mesirov J.P. (2004). Metagenes and molecular pattern discovery using matrix factorization. Proc. Natl. Acad. Sci. USA.

[bib8] Canudas S., Smith S. (2009). Differential regulation of telomere and centromere cohesion by the Scc3 homologues SA1 and SA2, respectively, in human cells. J. Cell Biol..

[bib9] Caron P., Aymard F., Iacovoni J.S., Briois S., Canitrot Y., Bugler B., Massip L., Losada A., Legube G. (2012). Cohesin protects genes against γH2AX Induced by DNA double-strand breaks. PLoS Genet..

[bib10] Carretero M., Ruiz-Torres M., Rodríguez-Corsino M., Barthelemy I., Losada A. (2013). Pds5B is required for cohesion establishment and Aurora B accumulation at centromeres. EMBO J..

[bib11] Countryman P., Fan Y., Gorthi A., Pan H., Strickland J., Kaur P., Wang X., Lin J., Lei X., White C. (2018). Cohesin SA2 is a sequence-independent DNA-binding protein that recognizes DNA replication and repair intermediates. J. Biol. Chem..

[bib12] Dorsett D., Ström L. (2012). The ancient and evolving roles of cohesin in gene expression and DNA repair. Curr. Biol..

[bib13] Fernández-Serra A., Rubio L., Calatrava A., Rubio-Briones J., Salgado R., Gil-Benso R., Espinet B., García-Casado Z., López-Guerrero J.A. (2013). Molecular characterization and clinical impact of TMPRSS2-ERG rearrangement on prostate cancer: comparison between FISH and RT-PCR. BioMed Res. Int..

[bib14] Forbes S.A., Beare D., Boutselakis H., Bamford S., Bindal N., Tate J., Cole C.G., Ward S., Dawson E., Ponting L. (2017). COSMIC: somatic cancer genetics at high-resolution. Nucleic Acids Res..

[bib15] Gelot C., Guirouilh-Barbat J., Le Guen T., Dardillac E., Chailleux C., Canitrot Y., Lopez B.S. (2016). The cohesin complex prevents the end joining of distant DNA double-strand ends. Mol. Cell.

[bib16] Grossman R.L., Heath A.P., Ferretti V., Varmus H.E., Lowry D.R., Kibbe W.A., Staudt L.M. (2016). Toward a shared vision for cancer genomic data. N. Engl. J. Med.

[bib17] Hastings P.J., Lupski J.R., Rosenberg S.M., Ira G. (2009). Mechanisms of change in gene copy number. Nat. Rev. Genet..

[bib18] Hill V.K., Kim J.S., Waldman T. (2016). Cohesin mutations in human cancer. Biochim. Biophys. Acta.

[bib19] Hopkins S.R., McGregor G.A., Murray J.M., Downs J.A., Savic V. (2016). Novel synthetic lethality screening method identifies TIP60-dependent radiation sensitivity in the absence of BAF180. DNA Repair (Amst.).

[bib20] Iannelli F., Galbiati A., Capozzo I., Nguyen Q., Magnuson B., Michelini F., D’Alessandro G., Cabrini M., Roncador M., Francia S. (2017). A damaged genome’s transcriptional landscape through multilayered expression profiling around in situ-mapped DNA double-strand breaks. Nat. Commun..

[bib21] Kakarougkas A., Ismail A., Chambers A.L., Riballo E., Herbert A.D., Künzel J., Löbrich M., Jeggo P.A., Downs J.A. (2014). Requirement for PBAF in transcriptional repression and repair at DNA breaks in actively transcribed regions of chromatin. Mol. Cell.

[bib22] Kim J.S., He X., Orr B., Wutz G., Hill V., Peters J.M., Compton D.A., Waldman T. (2016). Intact cohesion, anaphase, and chromosome segregation in human cells harboring tumor-derived mutations in STAG2. PLoS Genet..

[bib23] Kojic A., Cuadrado A., De Koninck M., Giménez-Llorente D., Rodríguez-Corsino M., Gómez-López G., Le Dily F., Marti-Renom M.A., Losada A. (2018). Distinct roles of cohesin-SA1 and cohesin-SA2 in 3D chromosome organization. Nat. Struct. Mol. Biol..

[bib24] Kong X., Ball A.R., Pham H.X., Zeng W., Chen H.Y., Schmiesing J.A., Kim J.S., Berns M., Yokomori K. (2014). Distinct functions of human cohesin-SA1 and cohesin-SA2 in double-strand break repair. Mol. Cell. Biol..

[bib25] Laddha S.V., Ganesan S., Chan C.S., White E. (2014). Mutational landscape of the essential autophagy gene BECN1 in human cancers. Mol. Cancer Res..

[bib26] Ladurner R., Kreidl E., Ivanov M.P., Ekker H., Idarraga-Amado M.H., Busslinger G.A., Wutz G., Cisneros D.A., Peters J.M. (2016). Sororin actively maintains sister chromatid cohesion. EMBO J..

[bib27] Li X., Baek G., Ramanand S.G., Sharp A., Gao Y., Yuan W., Welti J., Rodrigues D.N., Dolling D., Figueiredo I. (2018). BRD4 promotes DNA repair and mediates the formation of TMPRSS2-ERG gene rearrangements in prostate cancer. Cell Rep..

[bib28] Lin C., Yang L., Tanasa B., Hutt K., Ju B.G., Ohgi K., Zhang J., Rose D.W., Fu X.D., Glass C.K., Rosenfeld M.G. (2009). Nuclear receptor-induced chromosomal proximity and DNA breaks underlie specific translocations in cancer. Cell.

[bib29] Losada A. (2014). Cohesin in cancer: chromosome segregation and beyond. Nat. Rev. Cancer.

[bib30] Mani R.S., Amin M.A., Li X., Kalyana-Sundaram S., Veeneman B.A., Wang L., Ghosh A., Aslam A., Ramanand S.G., Rabquer B.J. (2016). Inflammation induced oxidative stress mediates gene fusion formation in prostate cancer. Cell Rep.

[bib31] Mazumdar C., Shen Y., Xavy S., Zhao F., Reinisch A., Li R., Corces M.R., Flynn R.A., Buenrostro J.D., Chan S.M. (2015). Leukemia-associated cohesin mutants dominantly enforce stem cell programs and impair human hematopoietic progenitor differentiation. Cell Stem Cell.

[bib32] Meisenberg C., Gilbert D.C., Chalmers A., Haley V., Gollins S., Ward S.E., El-Khamisy S.F. (2015). Clinical and cellular roles for TDP1 and TOP1 in modulating colorectal cancer response to irinotecan. Mol. Cancer Ther.

[bib33] Nik-Zainal S., Alexandrov L.B., Wedge D.C., Van Loo P., Greenman C.D., Raine K., Jones D., Hinton J., Marshall J., Stebbings L.A., Breast Cancer Working Group of the International Cancer Genome Consortium (2012). Mutational processes molding the genomes of 21 breast cancers. Cell.

[bib34] Osborne C.S. (2014). Molecular pathways: transcription factories and chromosomal translocations. Clin. Cancer Res..

[bib35] Polak P., Kim J., Braunstein L.Z., Karlic R., Haradhavala N.J., Tiao G., Rosebrock D., Livitz D., Kübler K., Mouw K.W. (2017). A mutational signature reveals alterations underlying deficient homologous recombination repair in breast cancer. Nat. Genet..

[bib36] Ran F.A., Hsu P.D., Wright J., Agarwala V., Scott D.A., Zhang F. (2013). Genome engineering using the CRISPR-Cas9 system. Nat. Protoc.

[bib37] Remeseiro S., Cuadrado A., Carretero M., Martínez P., Drosopoulos W.C., Cañamero M., Schildkraut C.L., Blasco M.A., Losada A. (2012). Cohesin-SA1 deficiency drives aneuploidy and tumourigenesis in mice due to impaired replication of telomeres. EMBO J..

[bib38] Sakaue-Sawano A., Kurokawa H., Morimura T., Hanyu A., Hama H., Osawa H., Kashiwagi S., Fukami K., Miyata T., Miyoshi H. (2008). Visualizing spatiotemporal dynamics of multicellular cell-cycle progression. Cell.

[bib39] Shanbhag N.M., Rafalska-Metcalf I.U., Balane-Bolivar C., Janicki S.M., Greenberg R.A. (2010). ATM-dependent chromatin changes silence transcription in cis to DNA double-strand breaks. Cell.

[bib40] Solomon D.A., Kim T., Diaz-Martinez L.A., Fair J., Elkahloun A.G., Harris B.T., Toretsky J.A., Rosenberg S.A., Shukla N., Ladanyi M. (2011). Mutational inactivation of STAG2 causes aneuploidy in human cancer. Science.

[bib41] Solomon D.A., Kim J.S., Bondaruk J., Shariat S.F., Wang Z.F., Elkahloun A.G., Ozawa T., Gerard J., Zhuang D., Zhang S. (2013). Frequent truncating mutations of STAG2 in bladder cancer. Nat. Genet..

[bib42] Tang J., Cho N.W., Cui G., Manion E.M., Shanbhag N.M., Botuyan M.V., Mer G., Greenberg R.A. (2013). Acetylation limits 53BP1 association with damaged chromatin to promote homologous recombination. Nat. Struct. Mol. Biol..

[bib43] Tomlins S.A., Rhodes D.R., Perner S., Dhanasekaran S.M., Mehra R., Sun X.W., Varambally S., Cao X., Tchinda J., Kuefer R. (2005). Recurrent fusion of TMPRSS2 and ETS transcription factor genes in prostate cancer. Science.

[bib44] Ui A., Nagaura Y., Yasui A. (2015). Transcriptional elongation factor ENL phosphorylated by ATM recruits polycomb and switches off transcription for DSB repair. Mol. Cell.

[bib45] Whelan G., Kreidl E., Wutz G., Egner A., Peters J.M., Eichele G. (2012). Cohesin acetyltransferase Esco2 is a cell viability factor and is required for cohesion in pericentric heterochromatin. EMBO J..

[bib46] Zhang N., Panigrahi A.K., Mao Q., Pati D. (2011). Interaction of Soronin protein with Polo-like kinase 1 mediates resolution of chromosomal arm cohesion. J. Biol. Chem..

