# Supplementary material for: Repression of Transcription at DNA Breaks Requires Cohesin throughout Interphase and Prevents Genome Instability
Source: Mol Cell. 2019 Jan 17;73(2):212–223.e7. doi: 10.1016/j.molcel.2018.11.001 (PMC6344341; doi:10.1016/j.molcel.2018.11.001)

Molecular Cell, Volume *73*

Supplemental Information

Repression of Transcription at DNA Breaks Requires Cohesin throughout Interphase and Prevents Genome Instability

Cornelia Meisenberg, Sarah I. Pinder, Suzanna R. Hopkins, Sarah K. Wooller, Graeme Benstead-Hume, Frances M.G. Pearl, Penny A. Jeggo, and Jessica A. Downs

SUPPLEMENTARY INFORMATION

TABLE S1. Mutational signatures associated with bladder cancers with and without mutations in SA2. Related to Figure 7. Bladder cancer samples were stratified based on SA2 mutational status (bld = wt SA2, stg = mutant STAG2) and analysed using NMMF (Figure 7A, B, see text and methods for details). These were compared to the signatures identified by (Alexandrov et al., 2015), and the assignments are listed below.

	Cluster 1	Cluster 2	Cluster 3	Cluster 4	Cluster 5	Cluster 6
**STAG2 wt**	bld_sig1	bld_sig2	bld_sig3	bld_sig4	bld_sig5	
**STAG2 mut**		stg_sig1	stg_sig3,5		stg_sig2	stg_sig4
**COSMIC**	nature 15	ature 1	gnature 13, 2	nature 16	nature 10	ature 3

TABLE S2: Antibodies, Related to STAR Methods

Antibody	Source	Identifier	Dilution	Host
**ARID2 (E-3)**	Santa Cruz	sc-166117	WB: 1:500	mouse
**ATM (D2E2)**	Cell Signlling	2873	WB: 1:1000	rabbit
**Baf180**	Millipore	ABE70	WB: 1:1000	rabbit
**BMI1**	Bethyl	A301-694A	WB: 1:1000	rabbit
**BRG1 (G7)**	Santa Cruz	sc-17796	WB: 1:500	mouse
**CENPF**	Bethyl	A301-611A	IS:1:250	rabbit
**CTCF**	Millipore	07-729	WB: 1:1000	rabbit
**Cyclin D1**	Neomarkers	RB-010-PO	IS:1:250	rabbit
**Esco2**	Abcam	ab86003	WB: 1:500	rabbit
**EZH2**	Cell Signaling	5246	WB: 1:1000	rabbit
**GFP (B2)**	Santa Cruz	sc-9996	WB: 1:1000	mouse
**H2A-K119ub (E5C5)**	Millipore	05-678	IS:1:250	mouse
**NIPBL**	Abcam	ab106768	WB: 1:1000	rat
**PDS5A**	Abcam	ab122352	WB: 1:1000	rabbit
**PDS5B**	Abcam	ab70298	WB: 1:1000	rabbit
**Rad21**	Abcam	ab992	WB: 1:1000	rabbit
**SMC3**	Abcam	ab155587	WB: 1:1000	rabbit
**SA1**	Abcam	ab4457	WB: 1:2000	goat
**SA2**	Abcam	ab4463	WB: 1:2000	goat
**WAPL**	Abcam	ab70741	WB: 1:1000	rabbit
**α-tubulin**	Abcam	ab7291	WB: 1:10,000	mouse
**γH2AX (JBW301)**	Millipore	05-636	IS:1:400	mouse
**Goat Anti-Rabbit HRP**	Agilent (Dako)	P044801-2	WB: 1:3000	
**Rabbit Anti-Mouse HRP**	Agilent (Dako)	P026002-2	WB: 1:3000	
**Rabbit Anti-Goat HRP**	Agilent (Dako)	P044901-2	WB: 1:3000	
**Goat Anti-Rat HRP**	Millipore	AP136P	WB: 1:3000	
**Goat Anti-Mouse FITC**	Sigma Aldrich	F0257-1ML	IS:1:300	
**Sheep Anti-rabbit Cy3**	Sigma Aldrich	C2306-1ML	IS:1:300	
**Goat Anti-Mouse Fluor 555**	ThermoFisher Scientific	A-21422	IS:1:300	

TABLE S3: siRNA sequences, Related to STAR Methods

siRNA	Source	Catalogue No / Sequence (5′-3′)
**ARID2 SMARTpool: ON-TARGETplus**	Dharmacon	L-026945-01-0005
**Baf180 SMARTpool: ON-TARGETplus**	Dharmacon	L-008692-01-0005
**BRG1 SMARTpool: ON-TARGETplus**	Dharmacon	L-010431-00-0005
**BRM SMARTpool: ON-TARGETplus**	Dharmacon	L-017253-00-0005
**CTCF SMARTpool: ON-TARGETplus**	Dharmacon	L-020165-00-0005
**Esco2 SMARTpool: ON-TARGETplus**	Dharmacon	L-025788-01-0005
**NIPBL SMARTpool: ON-TARGETplus**	Dharmacon	L-012980-00-0005
**NTC: Non-targeting pool: ON-TARGETplus**	Dharmacon	D-001810-10-20
**Rad21 SMARTpool: ON-TARGETplus**	Dharmacon	L-006832-00-0005
**SMC3 SMARTpool: ON-TARGETplus**	Dharmacon	L-006834-00-0005
**SA2 SMARTpool: ON-TARGETplus**	Dharmcon	L-021351-00-0005
**WAPL SMARTpool: ON-TARGETplus**	Dharmacon	L-026287-01-0005
**ATM**	Eurofins Genomics	CCAUGAAUCUAUUUAACGA
**NTC**	Eurogentec	UUCUUCGAACGUGUCACGU
**PDS5A (seq1)**	Eurogentec	GUGAUGCCUUCCUAAAUGA
**PDS5A (seq2)**	Eurogentec	GCUCCAUAUACUUCCCAUG
**PDS5B (seq1)**	Eurogentec	GCUCCUUACACAUCCCCUG
**PDS5B (seq2)**	Eurogentec	GAGACGACUCUGAUCUUGU
**Sororin**	Eurogentec	GCCUAGGUGUCCUUGAGCU
**SA1**	Eurofins Genomics	GUGAUGCCUUCCUAAAUGA
**SA2 (seq05)**	Eurogentec	GAAAUUUACUUGCAGCAUU
**SA2 (seq06)**	Eurogentec	GUAGAUGAUUGGAUAGAAU
**SA2 (seq07)**	Eurogentec	GGGAUUUAUUUGCUUGUAA
**SA2 (seq08)**	Eurogentec	CCACUGAUGUCUUACCGAA

TABLE S4: Primer sequences, Related to STAR Methods

Primer	Source	Sequence (5′ – 3′)
**Reporter transcript FWD**	Shanbhag et al., 2010	TCATTAGATCCTGAGAACTTCA
**Reporter transcript REV**	Shanbhag et al., 2010	TTTTGGCAGAGGGAAAAAGA
**TMPRSS2-CR-3F**	Li et al., 2018	CACCGTTCATTCACGATCCCTAACA
**TMPRSS2-CR-3R**	Li et al., 2018	AAACTGTTAGGGATCGTGAATGAAC
**ERG-CR-2F**	Li et al., 2018	CACCGGGATGGTAAACGGAGAGTGC
**ERG-CR-2R**	Li et al., 2018	AAACGCACTCTCCGTTTACCATCCC
**TMPRSS2_rtPCR FWD**	This paper	CTGGTGGCTGATAGGGGAT
**TMPRSS2_rtPCR REV**	This paper	GTCTGCCCTCATTTGTCGAT
**TMPRSS2:ERG FWD**	This paper	AGCGCGGCAGGAAGCCTTAT
**TMPRSS2:ERG REV**	Mani et al., 2016	CCGTAGGCACACTCAAACAACGA
**Actin_rtPCR FWD**	Lin et al., 2009	GCTCGTCGTCGACAACGGCTC
**Actin_rtPCR REV**	Lin et al., 2009	CAAACATGATCTGGGTCATCTTCTC
**cyclophilin A_rtPCR FWD**	This paper	CTGGACCCAACACAAATGGT
**cyclophilin A_rtPCR REV**	This paper	GCCTTCTTTCACTTTGCCAAAC
**Sororin_rtPCR FWD**	This paper	AGTCTCGCCAGTGGTGTGCT
**Sororin_rtPCR REV**	This paper	TTCAACCAGGAGATCAAACTGC
**GAPDH_rtPCR FWD**	This paper	ACATCGCTCAGACACCATG
**GAPDH_rtPCR REV**	This paper	TGTAGTTGAGGTCAATGAAGGG
**SA2 08_siRes_SDM_P1 FWD**	This paper	GGATGTGTGGCTTCCATTAATGTCTTACCGA
**SA2 08_siRes_SDM_P1 REV**	This paper	TCGGTAAGACATTAATGGAAGCCACACATCC
**SA2 08_siRes_SDM_P2 FWD**	This paper	GGCTTCCATTAATGAGTTACCGAAATTCTTTG
**SA2 08_siRes_SDM_P2 REV**	This paper	CAAAGAATTTCGGTAACTCATTAATGGAAGCC
**SA2_V181M_SDM FWD**	This paper	GTGAATTCATTGGCATGTTAGTACGGCAATGTC
**SA2_V181M_SDM REV**	This paper	GACATTGCCGTACTAACATGCCAATGAATTCAC
**SA2_S202L_SDM FWD**	This paper	GATGGATACAGTCATTTTACTTCTTACAGGATTG
**SA2_S202L_SDM REV**	This paper	CAATCCTGTAAGAAGTAAAATGACTGTATCCATC
**Rad21 HindIII_PCR FWD**	This paper	GCCAAGCTTGGTTCTACGCACATTTTGTTCTCAG

**Rad21 Sal1_PCR REV**	This paper	GCCGTCGACTTATATAATATGGAACCTTGGTCCAG
**SA2 HindIII_PCR FWD**	This paper	GGCAAGCTTCGATAGCAGCTCCAGAAATACCA
**SA2 Kpn1_PCR REV**	This paper	GCGGGTACCTTAAAACATTGACACTCCAAGAAC

SUPPLEMENTARY INFORMATION FIGURE LEGENDS

Figure S1. The centromere-specific cohesin complex is required for transcriptional repression at DNA double strand breaks. Related to Figure 1.
(A) Quantative PCR analysis of U2OS 263 IFII reporter transcript following indicated siRNA treatment, doxycycline-induced transcription (Tx) and mCherry-FokI DSB induction (DSB). Data represent SEM for n=5 biological replicates. **p,0.01, paired Student's *t* test. **(B)** Quantification of total EU intensity in U2OS cells treated with the indicated siRNA, data are represented as the mean +/- SD, n=4 biological replicates. **(C)** Western blot analysis of whole cell extracts prepared from BAF180 deletion (knockout; KO) and parental cells (U2OS) showing BAF180 deletion does not affect SA2 protein levels. **(D)** Quantification of EU intensity following laser microirradiation in BAF180 KO and parental U2OS cells. **(E)** Quantification of H2A K119ub foci in U2OS cells treated with the indicated siRNA, or irradiated BAF180 KO or parental U2OS cells 20 min following 1.5 Gy irradiation or no irradiation. 150 cells were counted per condition for each of n=3 biological replicates, and data are presented as the mean +/- SEM. *p<0.05, paired Student's *t* test. **(F)** Quantification of γH2AX foci in U2OS cells treated with the indicated siRNA following irradiation with 1.5 Gy. 36 cells counted per condition for each of n=3 biological replicates, and data are presented as the mean +/- SD. *p<0.05, paired Student's *t* test. **(G)** Western blot analysis of whole cell extracts prepared from samples treated as in (E). α-tubulin is used as a loading control.

Figure S2. Cohesin- and PBAF-dependent transcriptional repression at DNA double strand breaks occurs both in G1 and G2 phases. Related to Figure 2. (A) RFP-tagged Cdt1 expression is restricted to G1 and very early S phase and Geminin-GFP expression is restricted to late S and G2 (left panel, adapted from Thermofisher FUCCI Cell Cycle Sensor product information) and each was individually used to discriminate between cells in and outside of G1 or S/G2 phase (Cdt1 example shown in right panels). **(B)** Recruitment of RFP-tagged SA2 to laser microirradiation induced DNA damage in cells expressing GFP-tagged Geminin. SA2 recruitment in cells in GFP-positive (in late S/G2) and GFP-negative (outside of late S/G2) cells was similar. Data represents the relative mean signal intensity +/- SEM, n=3 biological replicates. **(C)** Western blot analysis of whole cell extracts prepared from cells transfected with no construct or eGFP-tagged BAF180 (used in Figure 2). α-tubulin is used as a loading control. **(D)** Western blot analysis of whole cell extracts prepared from cells transfected with no construct or eGFP-tagged SA2 (used in Figure 2). α-tubulin is used as a loading control. **(E)** Recruitment of eGFP-tagged STAG2 to *Fok*I-induced DSBs in the U2OS 265 reporter cell line. **(F)** Recruitment of eGFP-tagged Rad21 to laser microirradiation induced DNA damage. Data represents the relative mean signal intensity +/- SEM, n=1. **(G)** Representative images of Rad21 recruitment to laser microirradiation induced DNA damage (arrow). **(H)** Western blot analysis of whole cell extracts prepared from cells transfected with no construct or eGFP-tagged Rad21 (used in panels F and G). α-tubulin is used as a loading control. **(I)** Western blot analysis of whole cell extracts prepared from stable cell lines expressing shNTC or shBAF180 (Hopkins et al., 2016). α-tubulin is used as a

loading control. **(J)** Recruitment of eGFP-tagged STAG2 to laser microirradiation induced DNA damage in stable cell lines expressing shNTC or shBAF180. Data represents the relative mean signal intensity +/- SEM, n=2. **(K)** Recruitment of eGFP-tagged BAF180 to laser microirradiation induced DNA damage in siNTC- or siSA2- treated cell lines. Data represents the relative mean signal intensity +/- SEM, n=3 biological replicates.

Figure S3. CTCF is not required to promote transcriptional repression at DNA DSBs. Related to Figure 3. (A) Quantification of transcription in U2OS reporter cells with or without induction of the *Fok*I endonuclease treated with the indicated siRNA. Data are represented as the mean +/- SD. n=3. **(B)** Western blot analysis of whole cell extracts prepared from cells treated with siRNAs as in panel A. α-tubulin is used as a loading control. **(C)** Quantification of EU intensity following laser microirradiation following siRNA depletion of the indicated proteins. Data are represented as the mean +/- SEM for n=3 biological repeats.

Figure S4. Characterisation of individual siRNAs directed against SA2 for use in creating siRNA-resistant SA2 expression constructs. Related to Figure 4. (A) Western blot of whole cell extracts prepared from cells treated with the indicated siRNA. Individual siRNAs (05 through 08) were tested in parallel with the pooled siRNA (Mix) against SA2 to determine their silencing efficiency. α-tubulin is used as a loading control. **(B)** Quantification of transcription in U2OS reporter cells with or without induction of the *Fok*I endonuclease treated with the indicated siRNA. Individual siRNAs (05 through 08) were tested in parallel with the pooled siRNA (Mix) against SA2 to determine their impact on DNA DSB-induced

transcriptional silencing. Data are represented as the mean +/- SD. n=3 biological replicates. *p<0.05, **p<0.01, paired Student's *t* test. Expression constructs resistant to siRNA 08 were generated for use in the rescue experiments shown in Figure 4.

Figure S5. Androgen induced TMPRSS2 transcription is not substantially impaired in the absence of BAF180 or STAG2, and is repressed after irradiation. Related to Figure 5. (A, B) TMPRSS2 expression monitored by RT-qPCR in DHT-induced LNCaP cells treated with the indicated siRNA. Expression is normalised to Cyclophilin A (A) or actin (B). Data are represented by the mean +/- SD, n=4 (A) and n=2 (B) biological replicates. **(C)** TMPRSS2 expression monitored by RT-qPCR in LNCaP cells that were treated with DHT for 16 hours prior to irradiation with 10 Gy. Expression is normalised to Cyclophilin A. Data are represented by the mean +/- SD, n=7 biological replicates. ***p<0.001, paired Student's *t* test. **(D)** Translocations between TMPRSS2 and ERG (TMPRSS2:ERG) monitored by RT-qPCR in DHT-induced LNCaP cells treated with the indicated siRNA following irradiation with 0 or 10 Gy. Data are represented for n=3 biological replicates +/- SEM. *p<0.05, **p<0.01, ***p<0.001, paired Student's *t* test. **(E, F)** Western blot analysis of whole cell extracts prepared from cells treated with the indicated siRNA. α-tubulin is used as a loading control.

Figure S6. Depletion of cohesin or PBAF in U2OS cells leads to increased genome instability in actively transcribed genes. Related to Figure 6. (A) Experimental strategy for sample preparation. Following sample collection, genomic DNA was prepared and subjected to differential exome sequencing relative to untreated asynchronously growing U2OS cells. See text and methods for details. **(B)** Representative images of cells treated with siSA2 with (right panels) and without (left panels) irradiation and with (bottom panels) and without (top panels) DRB treatment. EU incorporation was used to monitor efficacy of DRB transcription inhibition. **(C)** FACS analysis of asynchronously growing U2OS cells (left panel) or U2OS cells treated as indicated following G1 cell cycle arrest using a double thymidine block. **(D)** Immunofluorescence using anti-γH2AX was used to monitor DNA DSB induction in irradiated or unirradiated cells treated with the indicated siRNAs. Images shown are from cells that were allowed to recover for 30 min following irradiation with 10 Gy and an unirradiated control (UT). **(E)** Table showing quality control data for exome sequencing. **(F)** Western blot analysis of whole cell extracts prepared from cells transfected with FLAG-tagged Cas9 + TMPRSS2 and ERG guide RNAs (Cas9-guide T/E) as illustrated in Figure 6C. DHT added to cell cultures at the same time as plasmid transfection in lanes with ‘+’, and at 16hr post-transfection in lanes with ‘*’. Cas9 expression is unaffected by DHT treatment. **(G)** Western blot analysis showing knockdown efficiency at Day3 (the point of Cas9 transfection) in LNCaP cells treated with the indicated siRNA.

S1 (related to Fig 1)

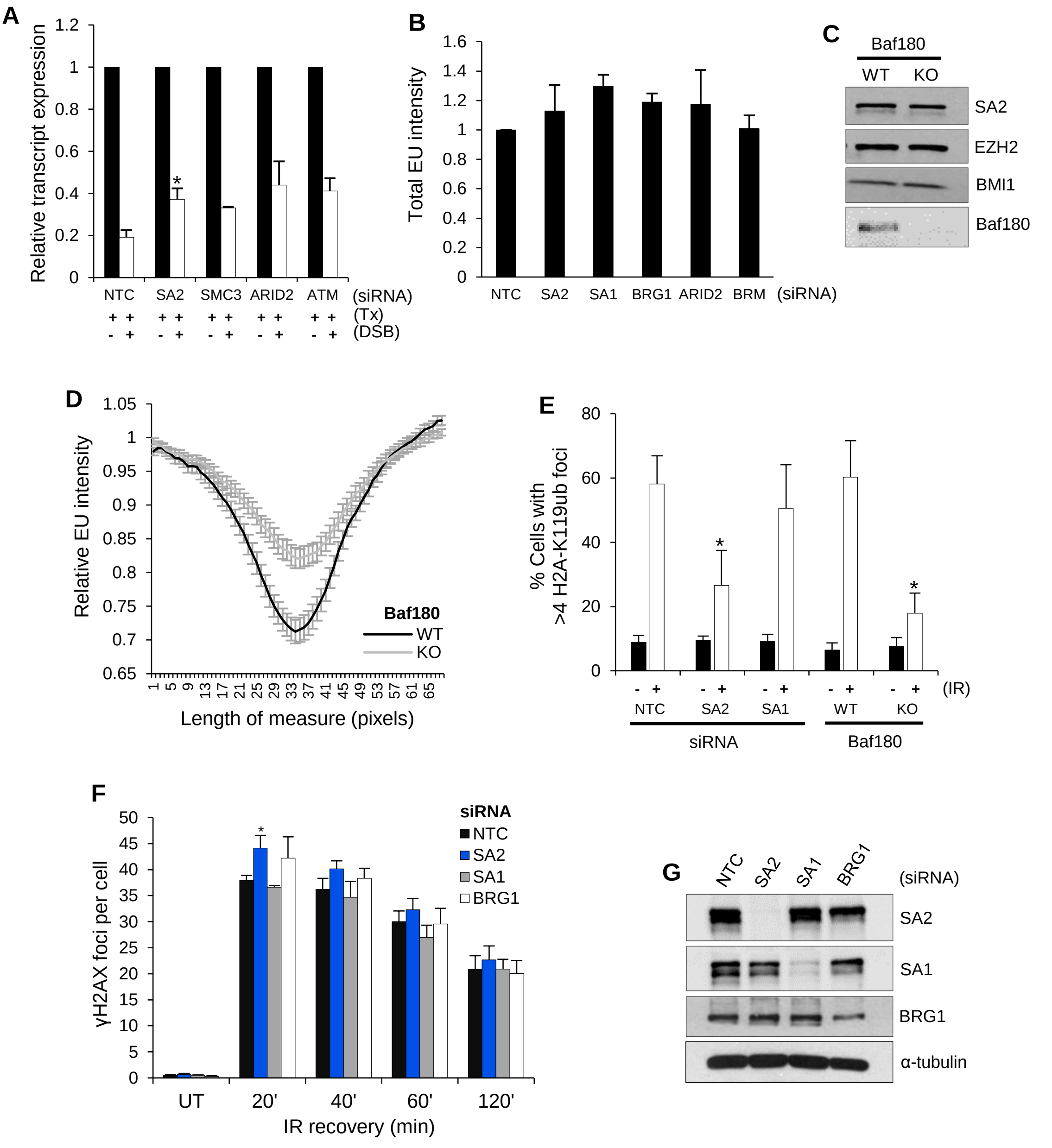

S2 (related to Fig 2)

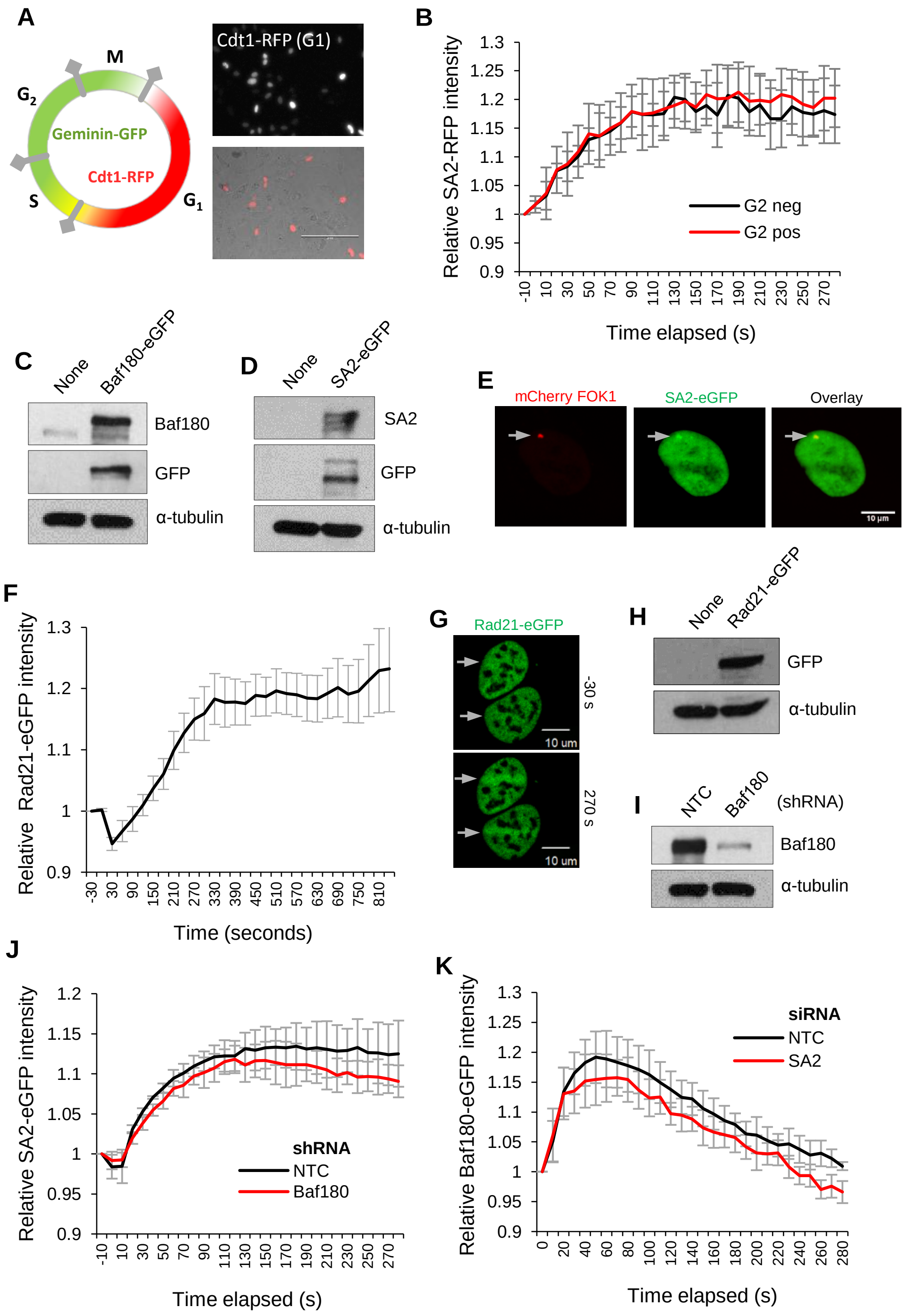

S3 (related to 3)

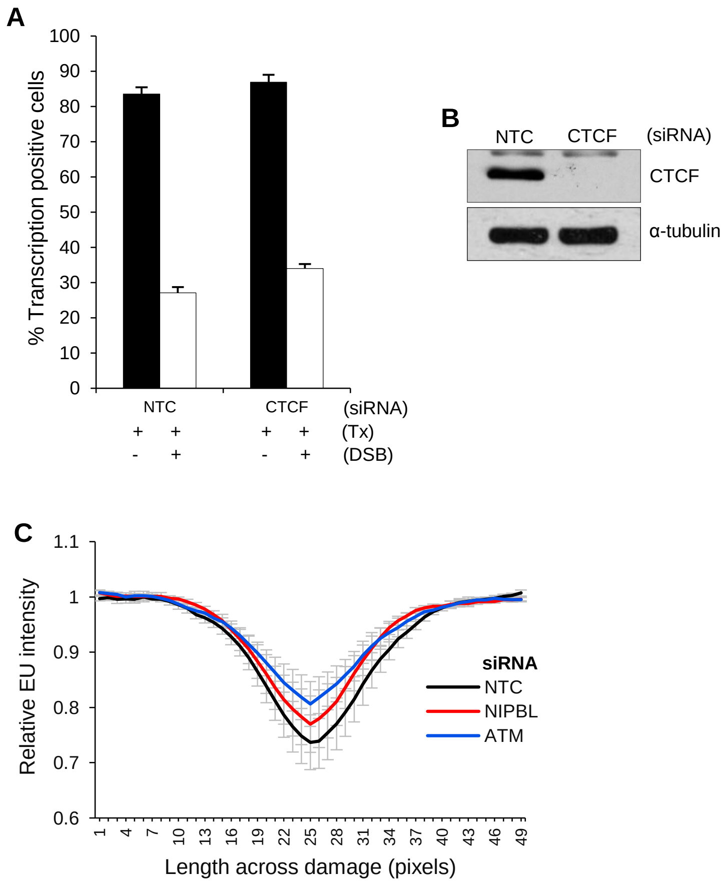

S4 (related to Fig 4)

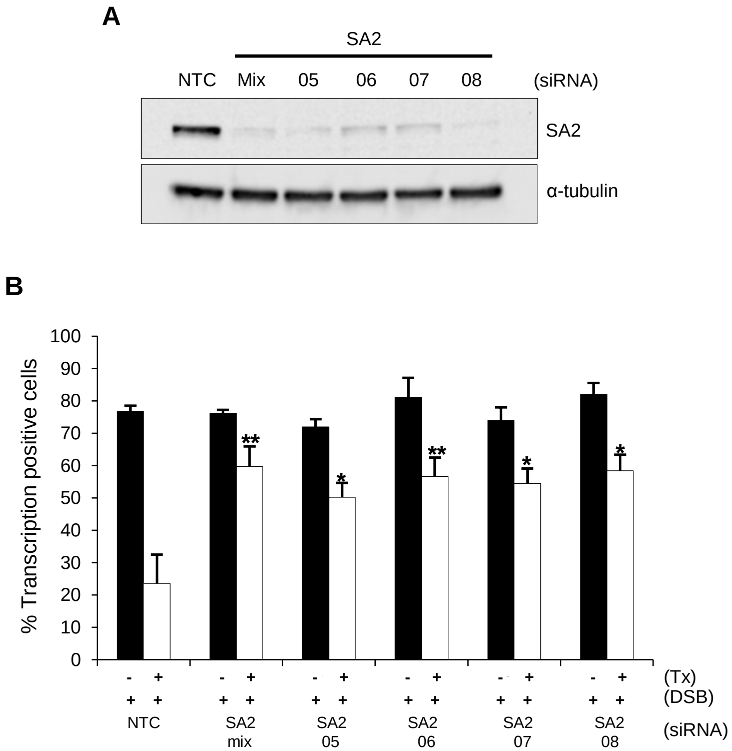

S5 (related to Fig 5)

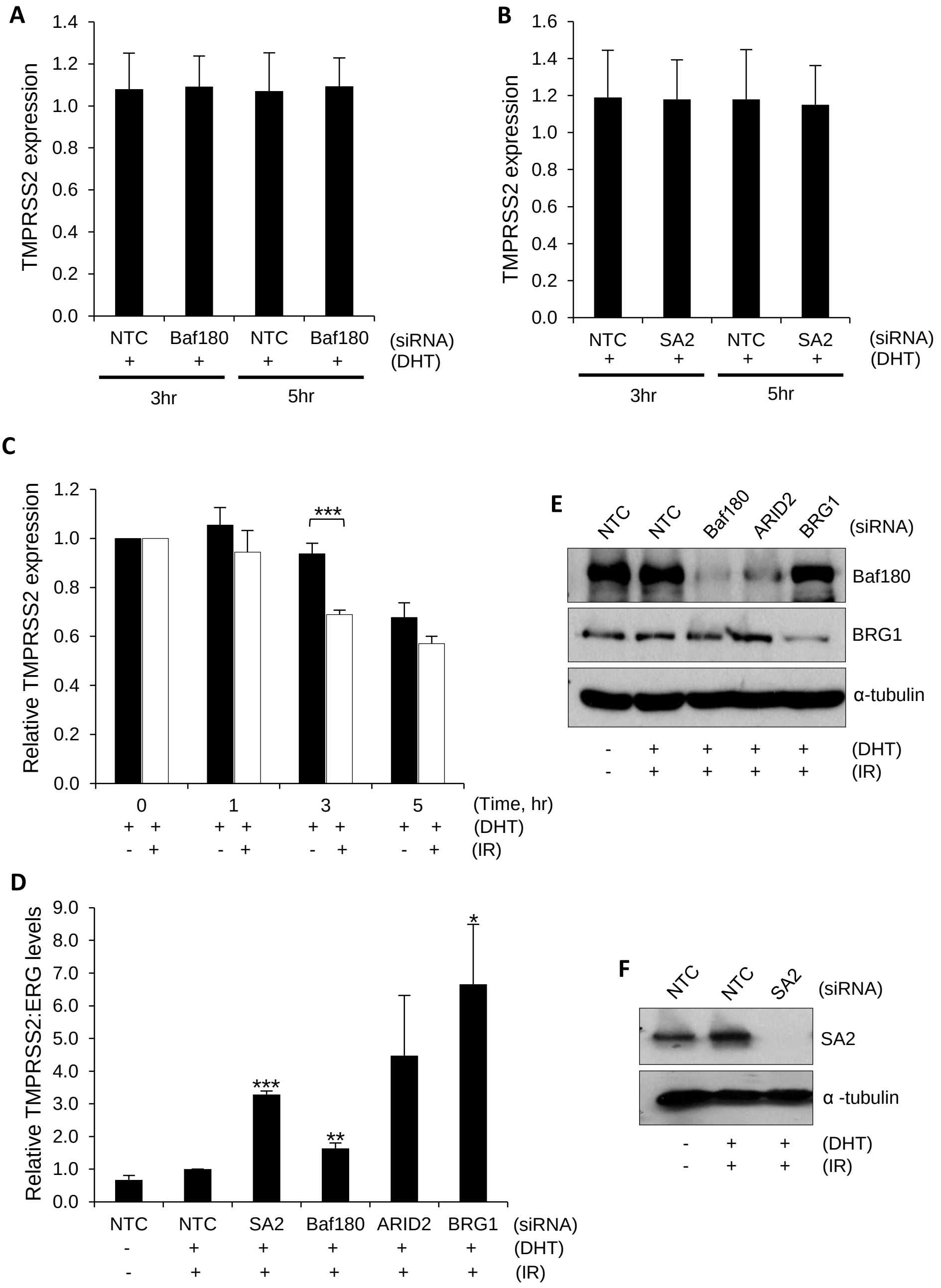

S6 (related to Fig 6)

A

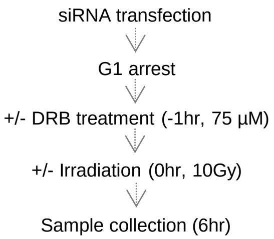

C

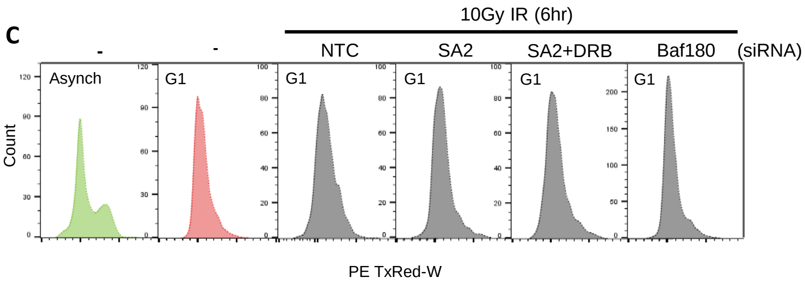

B

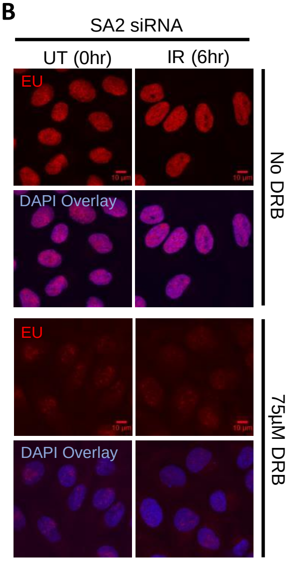

D

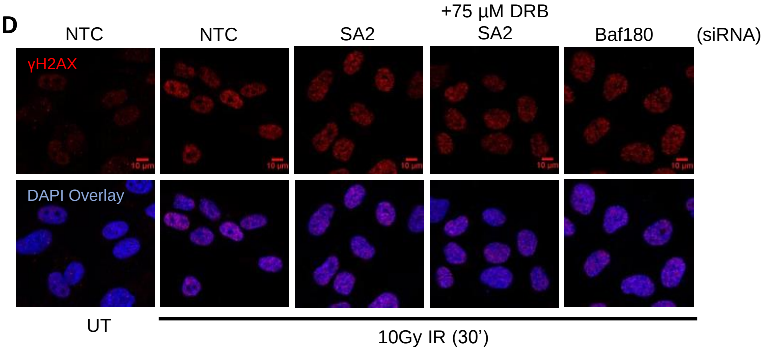

E

Sample Name	Total Unique Reads	% Reads Mapped	% Reads on Target	% of Duplicates	Median Depth	Mean Coverage
Asynch_UT	95,658,434	94.27	68.29	5.39	78	92
NTCsi_UT	95,566,183	94.53	69.79	5.41	80	94
NTCsi_10Gy	95,490,278	94.38	69.88	5.23	80	94
SA2si_UT	113,244,833	94.33	69.50	5.64	94	111
SA2si_10Gy	114,054,732	94.51	68.01	5.14	92	109
SA2si_10GyDRB	108,036,400	94.47	67.90	4.99	87	103
Baf180si_UT	100,240,936	94.34	68.62	5.28	81	97
Baf180si_10Gy	95,927,091	94.32	67.83	5.10	77	91

F

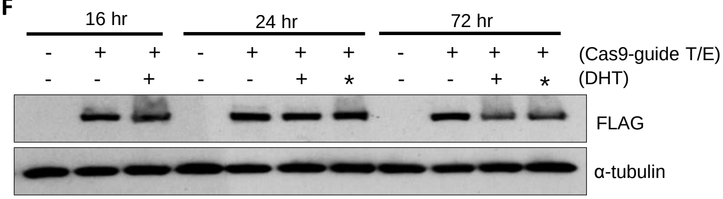

G

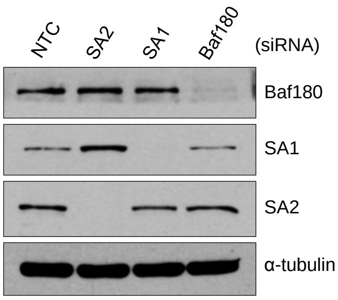

Supplement: Document S1. Figures S1–S6 and Tables S1–S4 [file mmc1.pdf]
